# Supplementary material for: Many plants naturalized as aliens abroad have also become more common within their native regions
Source: Nat Commun. 2025 Sep 5;16:8227. doi: 10.1038/s41467-025-63293-6 (PMC12413455; doi:10.1038/s41467-025-63293-6)
Supplement: Supplementary file 1 — Supplementary Information [file 41467_2025_63293_MOESM1_ESM.pdf]

Many plants naturalized as aliens abroad have also become more common within their native regions

Rashmi Paudel, Trevor S. Fristoe, Nicole L. Kinlock, Amy J.S. Davis, Weihan Zhao, Hans Van Calster, Milan Chytrý, Jiří Danihelka, Guillaume Decocq, Luise Ehrendorfer - Schratt , Kun Guo, Wen-Yong Guo, Zdeněk Kaplan, Simon Pierce, Jan Wild, Wayne Dawson, Franz Essl, Holger Kreft, Jan Pergl, Petr Pyšek, Marten Winter & Mark van Kleunen

* Rashmi Paudel

Email: rashmi.paudel@uni-konstanz.de

This PDF file includes:

Supplementary methods

Figures S1 to S12

Tables S1 to S36

SI References

Supplementary methods

Details on occupancy-change data for the ten native regions

For Austria, we obtained data on the occupancy of 2419 native species for the periods before and after 1990 from the "Floristische Kartierung Österreichs" [1]. Information on the native status of the species was provided by the source database.

For the Czech Republic, we obtained data on the occupancy of 1835 native species for the periods before and after 2000 from the Pladias-database of the Czech Flora and Vegetation (http://www.pladias.cz) [2]. Information on the native status of the species was provided by the source database.

For southeastern Denmark, we extracted data on the occupancy of 921 native species for the periods 1857-1883 and 2015 from Nielsen et al. [3]. Nielsen et al. [3] provide regional abundance and the total numbers of grid cells, separately for eleven subregions in southeastern Denmark. For each species, abundance was calculated by dividing the number of grid cells occupied by the species by the total number of grid cells for the subregion. From these data, we back calculated the numbers of grid cells occupied by a species in each subregion, and then combined them across the 11 subregions to get one single occupancy value. The occupancy data do therefore not cover all of southeastern Denmark but the combined area of the 11 subregions. Information on the native status of the species was provided by the original publication.

For Flanders (including the capital region of Brussels), we obtained data on the occupancy of 862 native species for the periods 1939–1971 and 1972–2004 from Van Landuyt et al. [4]. Information on the native status of most species was provided by the original publication, and, for the few cases where the native status was not clear, we checked the native

status in the Plants of the World Online database (POWO; https://powo.science.kew.org/; accessed on May 2023).

For Germany, we extracted data on occupancy of 1715 native species for the periods 1960-1987 and 1997-2017 from Eichenberg et al. [5]. Eichenberg [5] did not provide the observed occupancies. Instead estimates of occupancies based on the FREquency SCAling using Local Occupancy (FRESCALO) algorithm [6], which supposedly reduces potential taxonomic and spatial reporting biases, were provided. Information on the native status of the species was provided by the original data source.

For Great Britain, also including the Isle of Man and the Channel Islands, we obtained data on the occupancy of 1357 native species for the periods 1970-1986, 1987-1999 and 2000-2019 from the Plant Atlas 2020 [7]. To cover the largest possible time span, we used the periods 1970-1986 and 2000-2019 for the main analysis. However, we also did the analysis for the periods 1987-1999 and for the periods 2000-2019 and 1970-1986 and 1987-1999. These additional analyses showed largely similar patterns **(Table S26, S27)**. Furthermore, the Plant Atlas 2020 distinguishes between grid cells where the species is supposed to be native and those where it is considered non-native. For the main analysis, we did not distinguish between native and non-native grid cells. However, when we excluded the grid cells that were assigned as being non-native occurrences, the results were very similar **(Table S28)**. Information on the native status of the species was provided by the original data source.

For Ireland, we obtained data on the occupancy of 911 native species for the periods 1970-1986, 1987-1999 and 2000-2019 from the Plant Atlas 2020 [7]. Because the period 1970-1986 was not intensively recorded in Ireland [8], we used the periods 1987-1999 and 2000-2019 for the main analysis. However, we also did the analysis for the periods 1970-1986 and 2000-2019 and for the periods 1970-1986 and 1987-1999. These additional analyses showed

largely similar patterns **(Table S29, S30)**. Furthermore, similar to Great Britain, the Plant Atlas 2020 distinguishes between grid cells where the species is supposed to be native and those where it is considered non-native. For the main analysis, we did not distinguish between native and non-native grid cells. However, when we excluded the grid cells that were assigned as being non-native occurrences, the results were very similar **(Table S31)**. Information on the native status of the species was provided by the original data source.

For the Netherlands, we initially extracted data on the occupancy of 1115 native species for the periods pre-1990 and 1990-2022, and later, to see how robust the results are, also for the periods pre-2000 and 2000-2024 from the Nationale Databank Flora en Fauna [9]. For the main analysis, we used the periods pre-1990 and 1990-2022, but results for the periods pre-2000 and 2000-2024 were very similar **(Table S32)**. Information on the native status of the species was provided by the original database.

For Switzerland, we obtained data on the occupancy of 2307 native species for the periods pre-2000 and 2000-2022 from InfoFlora (The National Data and Information Center on the Swiss Flora) [10]. Information on the native status of the species was provided by the original database.

For Thiérache, which is located in the Northeast of the Aisne department in northern France, we obtained data on the occupancy of 775 native species for the periods 1880-1900 and 1957-2005 from Van Calster et al. [11]. Information on the native status of the species was provided by the original data source.

Data on woodiness of the species and statistical analysis

Whether species are likely to increase in occupancy in their native region and successfully naturalized elsewhere should ultimately depend on their traits. Unfortunately, data are still very sparse for most traits [12]. However, one of the traits that is available for many species is woodiness, which is an important trait as it provides some insights about growth form and the habitat preferences of the species. We therefore searched for data on woodiness of the 3920 species included in our 10 datasets. To obtain information on woodiness of species, we first collected data on their growth forms from Taylor et al. [13], Henniges et al. [14], the World checklist of vascular plants (WCVP, version 11; https://powo.science.kew.org/, accessed in April 2023) and the GIFT database [15] (https://gift.uni-goettingen.de/, version 3.2, extracted using the GIFT package [16] in R). Then, species classified as trees and shrubs were assigned as woody, while herbs, aquatic plants and ferns were categorized as non-woody. For species without growth form data in these datasets, we assigned woodiness based on additional literature, specifically Luo et al. [17] and Zanne et al. [18]. Finally, for the species that are not present in any of the above-mentioned sources, we did internet searches. Our compilation of woodiness data for the 3920 species, along with information on the data sources used, is available in the repository (Figshare; https://doi.org/10.6084/m9.figshare.25487209).

To test whether the inclusion of woodiness changed the results for the associations of global naturalization success with early occupancy and occupancy change in each of the 10 native regions, we reran the hurdle models described in the main text and added woodiness and its interactions with early occupancy and occupancy change as predictors. The results are shown in **Table S1, S2-S11**. Additionally, to test how woodiness is associated with early occupancy and occupancy change, we conducted separate Mann-Whitney U-test and two-sided t-test. In each model woodiness was used as a predictor variable, while early occupancy and

occupancy change, were response variables respectively. The results of these tests are shown in **Table S22 and S23**.

Data on native range size

To assess how occupancy in the 10 native regions correlates with size of the native range **(Table S35)**, we extracted for each of the 3920 species in the 10 native regions, the number of regions in which it is native according to POWO (https://powo.science.kew.org/, accessed in April 2023). The regions correspond to botanical countries (TDWG3 regions, n = 397) of the World Geographical Scheme for Recording Plant Distributions, previously called the Taxonomic Databases Working Group (TDWG) [19]. Data on the total number of native botanical countries for each of the 3920 species is available in the repository (Figshare; https://doi.org/10.6084/m9.figshare.25487209).

Data on Grime's CSR strategy and Ellenberg environmental indicator values and statistical analysis

To explore features of the species that had high early occupancy during the earlier time period and that since then had further increased their occupancy, we conducted additional analyses using Grime's CSR strategy [20] and Ellenberg environmental indicator values for light, moisture, nutrients and temperature [21]. Data on Grime's CSR strategy was obtained from [22], covering 2,126 of the 3,920 species included in our 10 datasets. The C-, S- and R- scores had been calculated from data on specific leaf area, leaf area and leaf dry matter content, following the PCA-based approach of [23]. For Ellenberg indicator values, we used the harmonized European-scale dataset published by Tichý et al. [24], supplemented with data from the Pladias database of the Czech Flora and Vegetation (http://www.pladias.cz) [25]. In

total, Ellenberg indicator values were available for 2,724 species from Tichý et al. [24] and for an additional 170 species from Pladias [25].

We then classified a species as widespread and expanding in a region if it had an early occupancy higher than the median of early occupancy of that region and a positive occupancy change. To test whether this group significantly differed from all other species, in terms of CSR strategy and Ellenberg indicator values, we performed non-parametric Mann-Whitney tests. In each model, the predictor was the two species groups—widespread and expanding vs. all others—, while CSR and Ellenberg indicator values were response variables. The results of these tests with CSR strategies are shown in **Table S33 and Fig. S4-Fig. S6** and with Ellenberg indicators are shown in **Table S 34 and Fig. S7-Fig. S10.**

Supplementary Figures and Tables

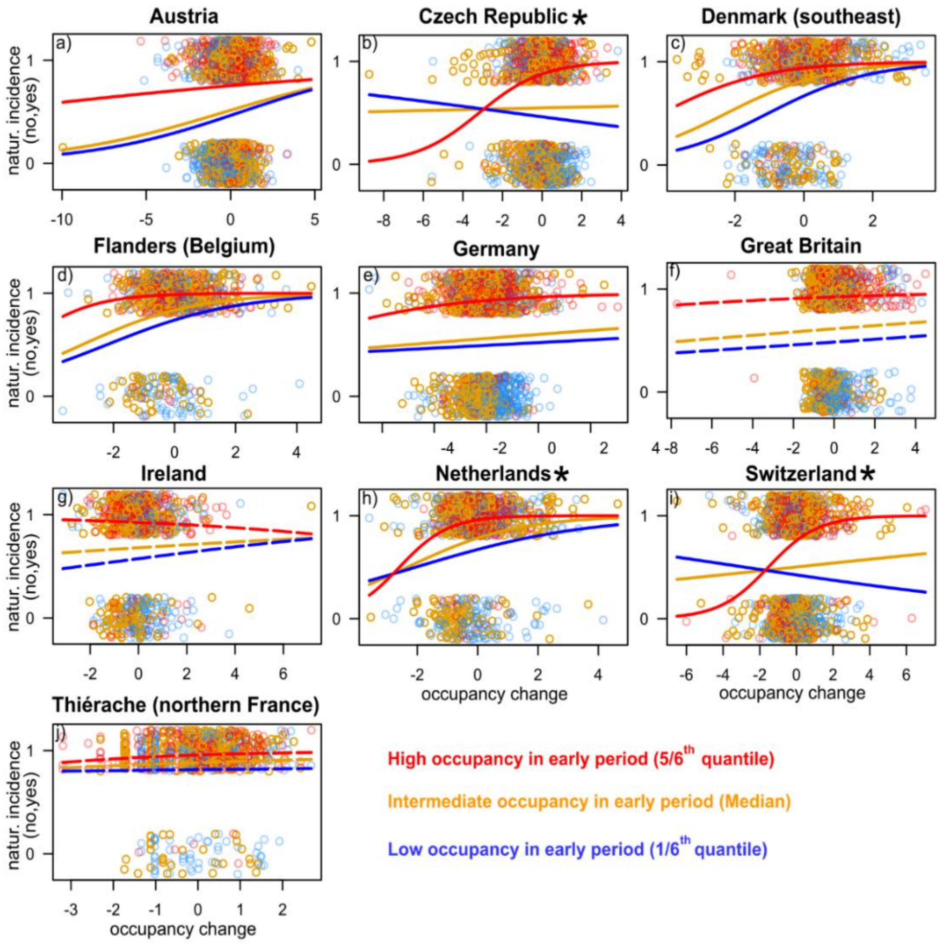

Fig. S1. Predicted relationships between naturalization incidence (i.e. whether or not a species has become naturalized) and the occupancy-change index for 10 native regions (a-j). These results are based on the Bernoulli parts of the hurdle models. To illustrate how naturalization incidence depends on early occupancy in the native region, the data points are coloured according to whether they are in the upper, middle or lower third of the early occupancy distribution. Accordingly, the predicted relationships are plotted for early occupancy values set equal to the 5/6th quantile, the median and the 1/6th quantile. Significant relationships between naturalization incidence and occupancy change are plotted with solid lines, and non-significant relationships are plotted with dashed lines. The regions for which the interaction between early occupancy and occupancy change was significant are marked with an asterisk (*) next to the region names. Source data are provided as a Source Data file.

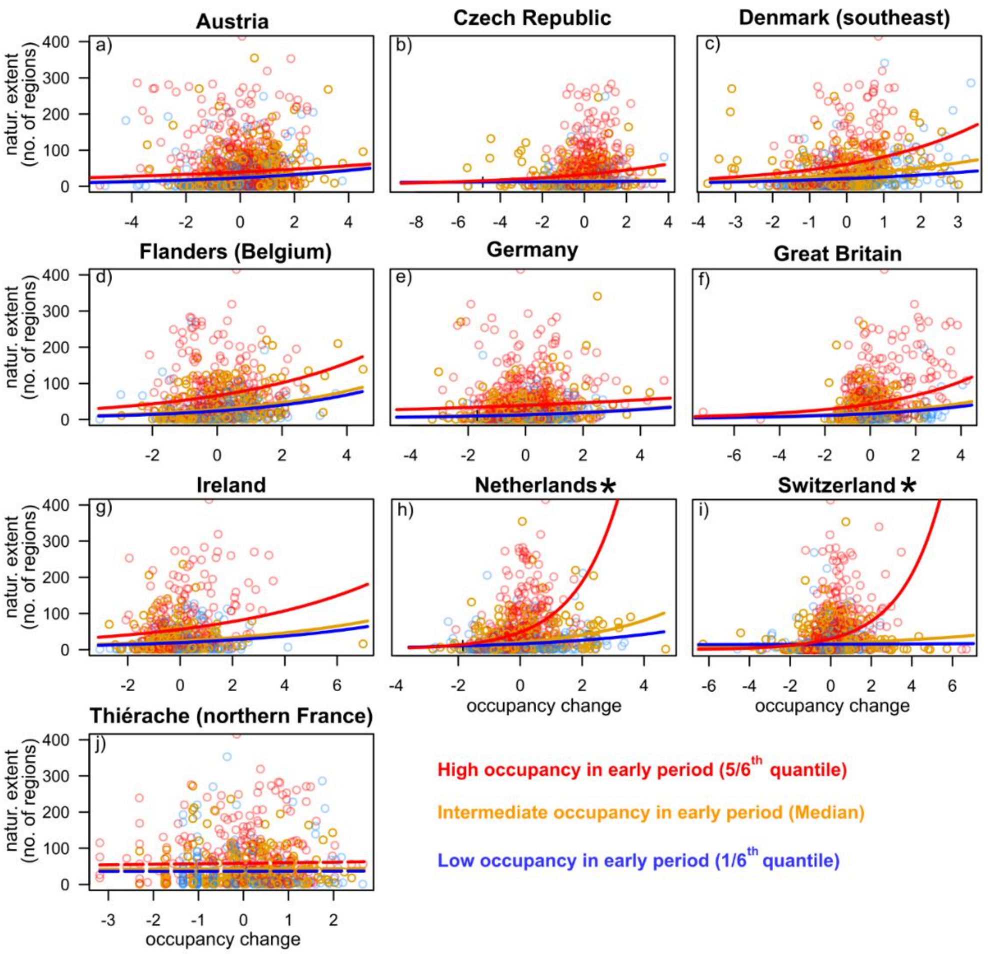

Fig. S2. Predicted relationships between naturalization extent (number of regions where a species has become naturalized) and the occupancy-change index in10 native regions (a-j). These results are based on the zero-truncated count parts of the hurdle models. To illustrate how naturalization extent depends on early occupancy in the native regions, the data points are coloured according to whether they are in the upper, middle or lower third of the early occupancy distribution. Accordingly, the predicted relationships are plotted for early occupancy values set equal to the $5/6^{th}$ quantile, the median and the $1/6^{th}$ quantile. Significant relationships between naturalization extent and occupancy change are plotted with solid lines, and non-significant relationships are plotted with dashed lines. The regions for which the interaction between early occupancy and occupancy change was significant are marked with an asterisk (*) next to the region names. Source data are provided as a Source Data file.

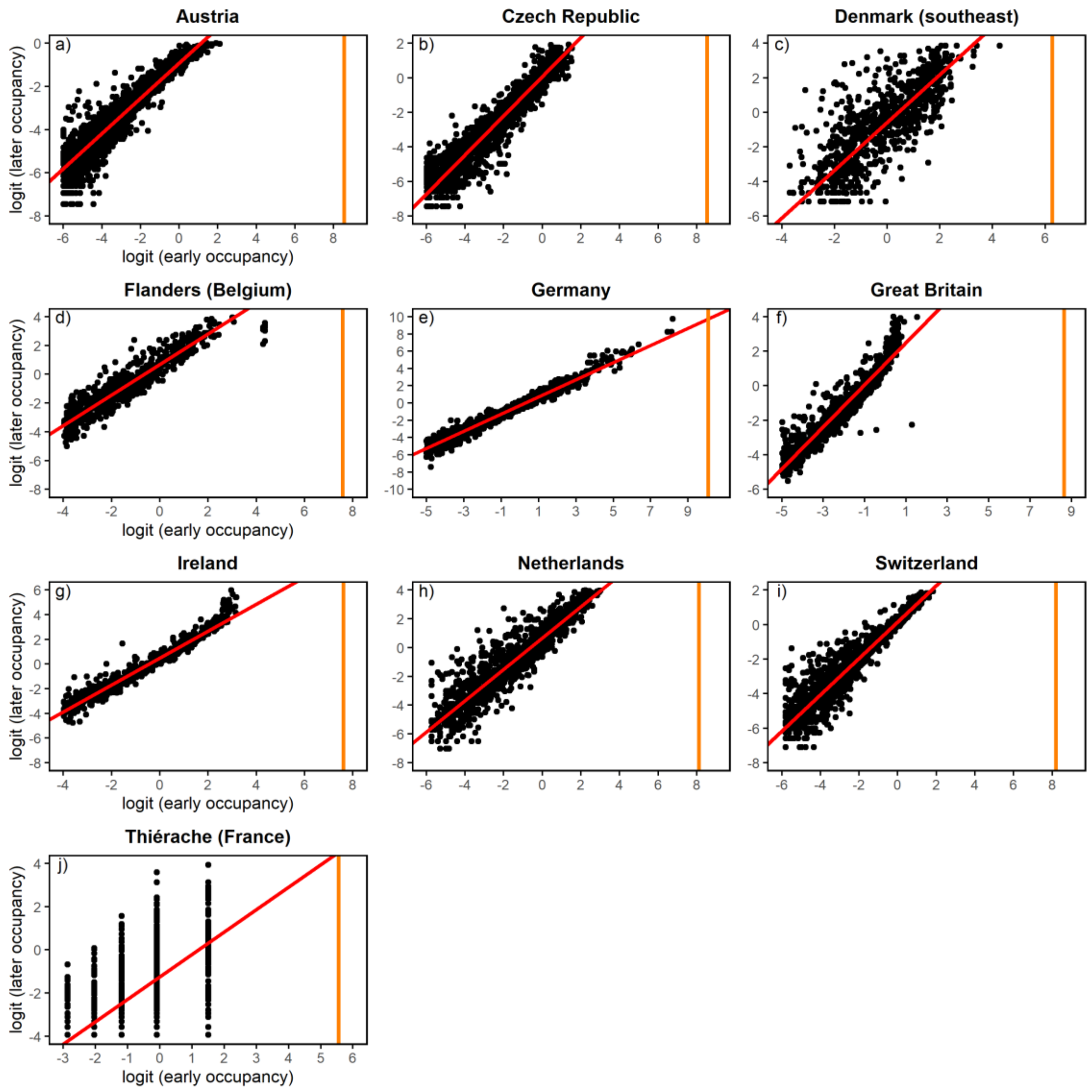

Fig. S3. **Weighted linear regression plots of the logit-transformed occupancy in the later period vs the logit-transformed occupancy in the early period for each of the 10 native regions (a-j)**. The occupancy-change index for each species corresponds to its deviation from the weighted regression line (i.e. the standardized residual; Telfer et al. [26]). Note that for Thiérache, there were only five possible occupancy values in the early period, because the grid cell numbers were estimated from verbal descriptions. The orange vertical lines indicate the logit of the total number of grid cells in each region. Source data are provided as a Source Data file.

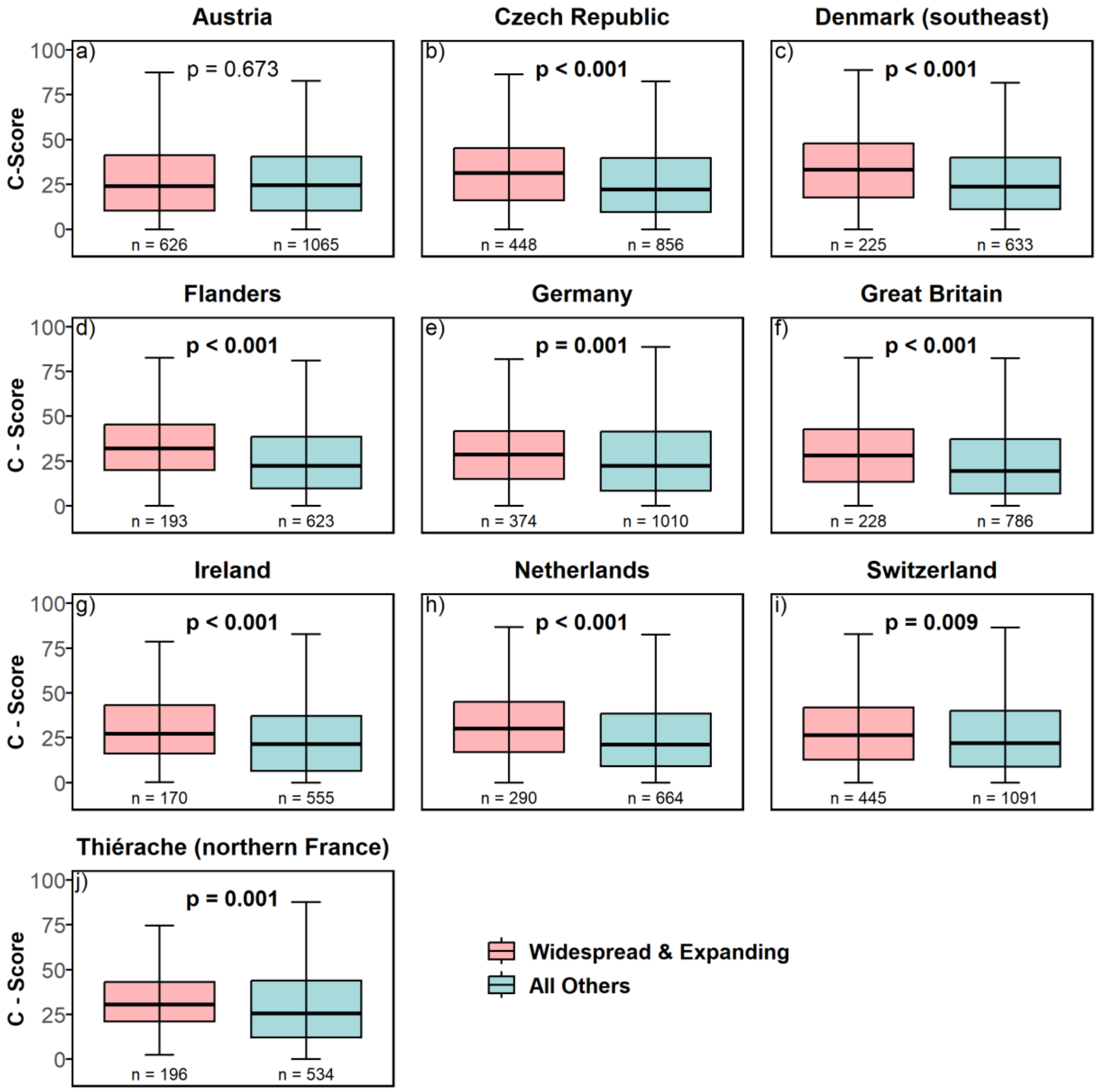

Fig. S4. Comparing scores for competitive strategies between two species groups: "widespread and expanding" vs. all others for each of the 10 native regions (a-j). *P* values are from the Mann-Whitney U test (see table S35), with significant values highlighted in bold. Sample sizes for each group in each native region are indicated by ‘n’ at the bottom of each box. Source data are provided as a Source Data file.

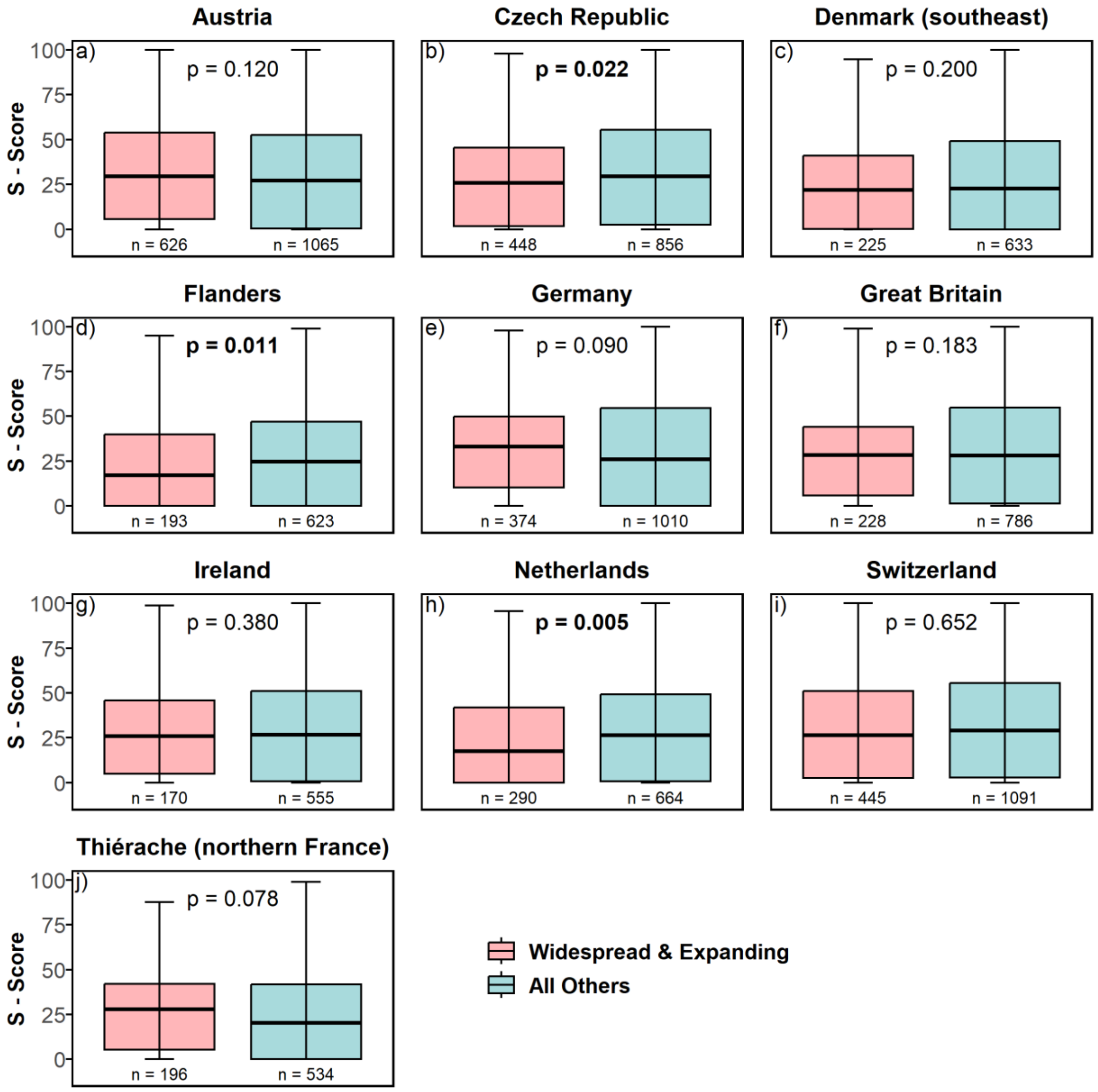

Fig. S5. Comparing scores for Stress-tolerator strategies between two species groups: "widespread and expanding" vs. all others for each of the 10 native regions (a-j). *P* values are from Mann-Whitney U test (see table S35), with significant values highlighted in bold. Sample sizes for each group in each native region are indicated by 'n' at the bottom of each box. Source data are provided as a Source Data file.

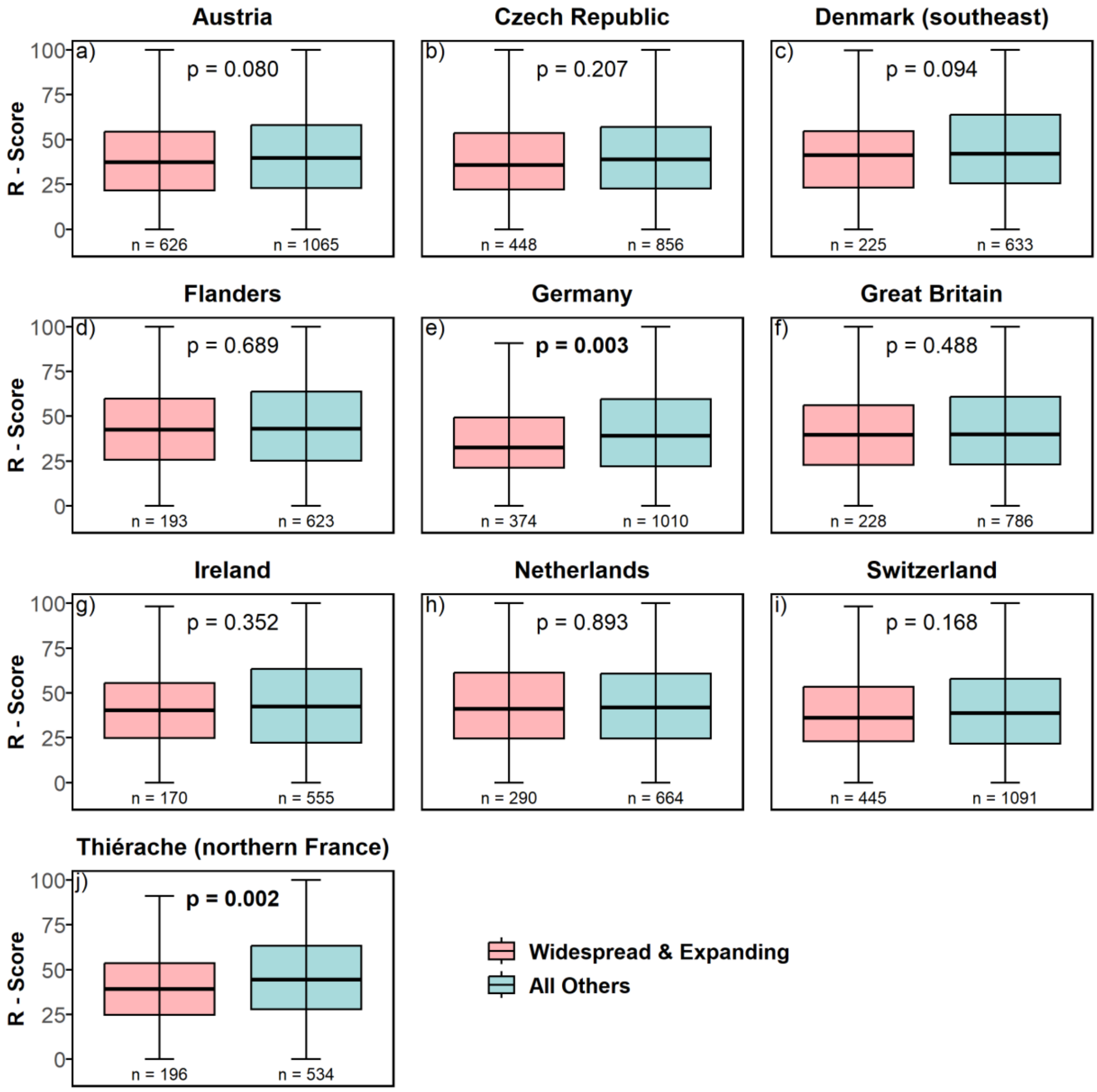

Fig. S6. Comparing scores for Ruderal strategies between two species groups: "widespread and expanding" vs. all others for each of the 10 native regions (a-j). *P* values are from Mann-Whitney U test (see table S35), with significant values highlighted in bold. Sample sizes for each group in each native region are indicated by ‘n’ at the bottom of each box. Source data are provided as a Source Data file.

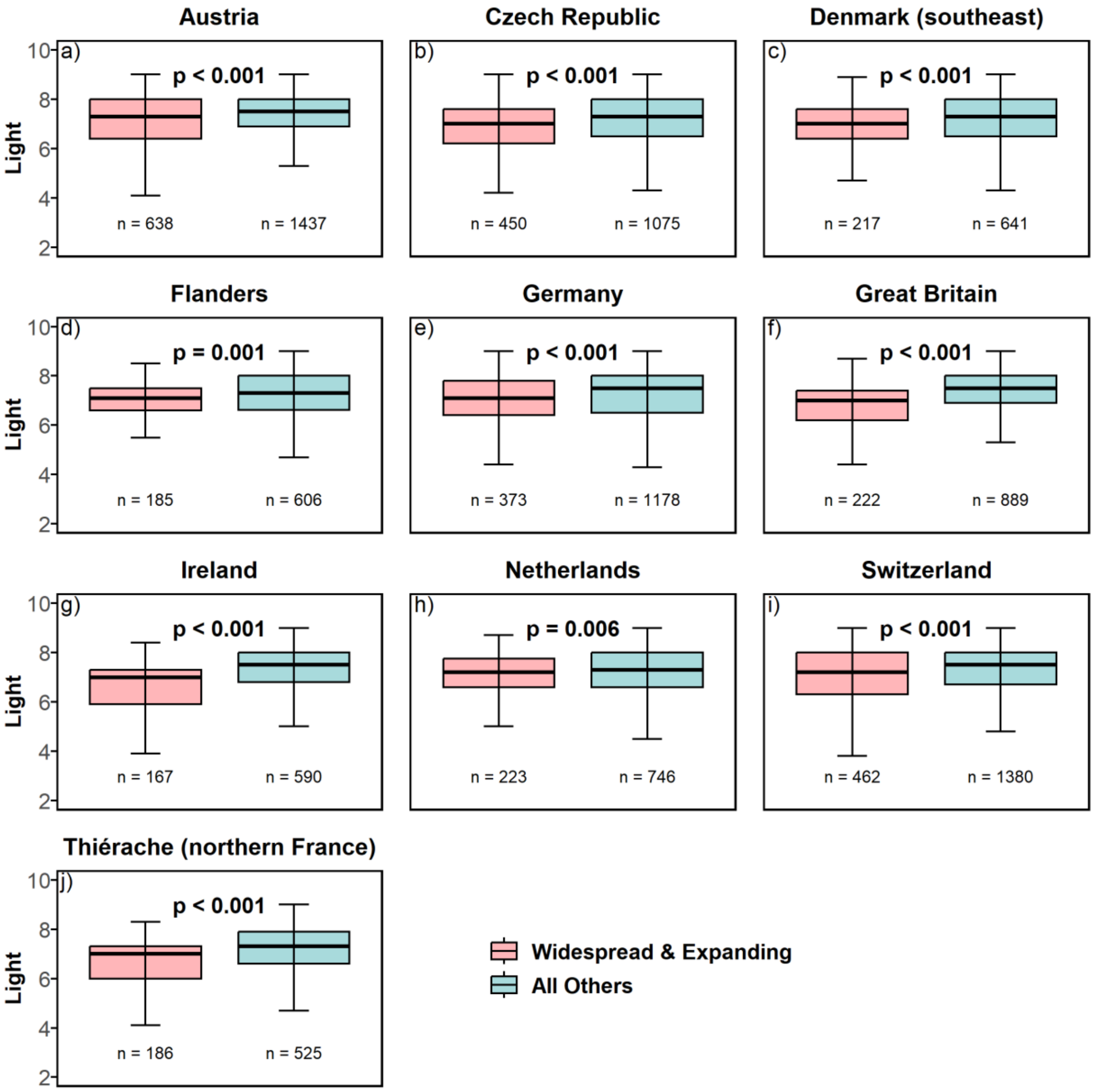

Fig. S7. Comparing Ellenberg indicator values for light between two species groups: "widespread and expanding " vs. all others for each of the 10 native regions (a-j). *P* values are from Mann-Whitney U test (see table S36), with significant values highlighted in bold. Sample sizes for each group in each native region are indicated by 'n' at the bottom of each box. Source data are provided as a Source Data file.

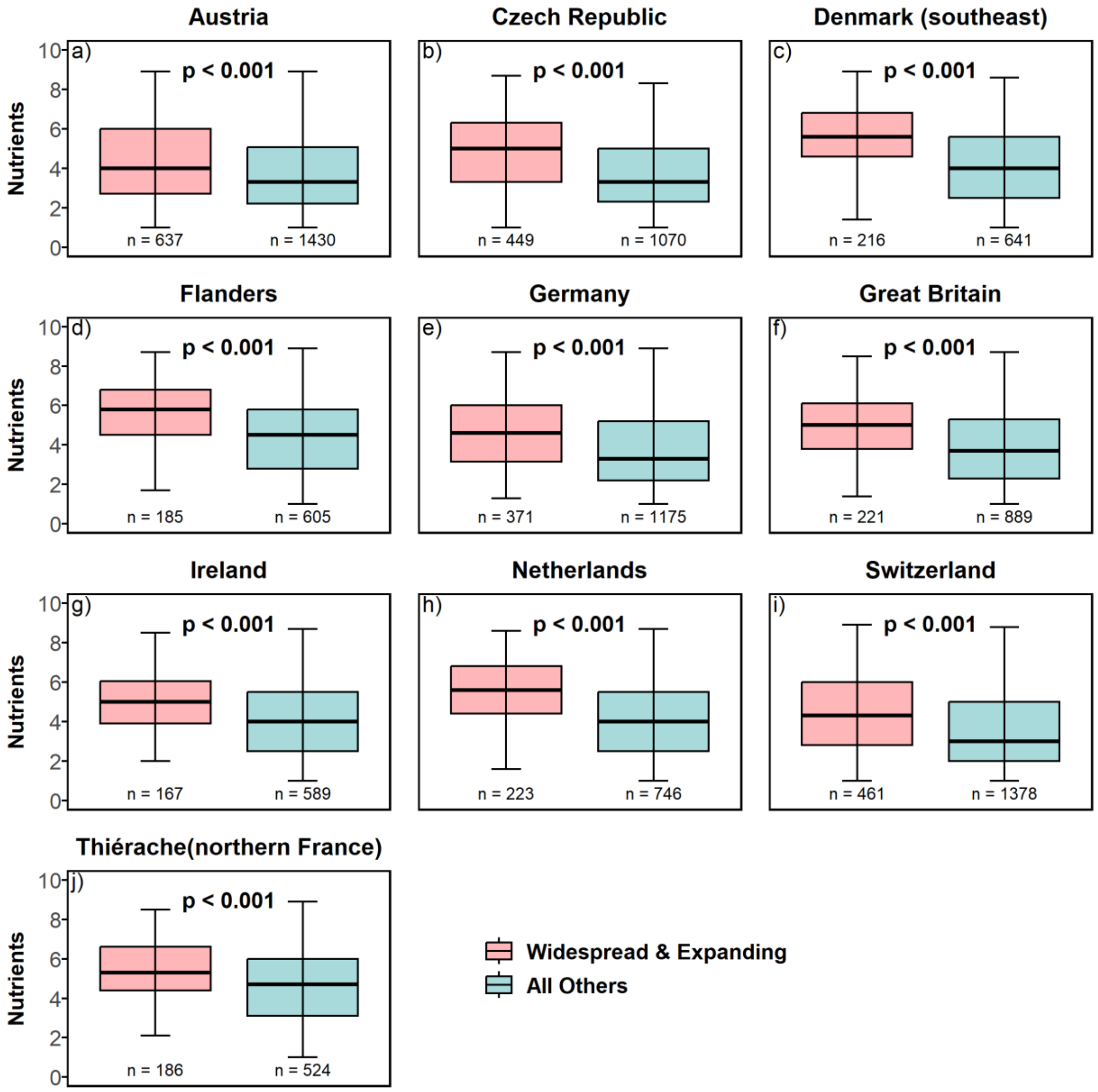

Fig. S8. Comparing Ellenberg indicator values for nutrients between two species groups: "widespread and expanding " vs. all others for each of the 10 native regions (a-j). *P* values are from Mann-Whitney U test (see table S36), with significant values highlighted in bold. Sample sizes for each group in each native region are indicated by ‘n’ at the bottom of each box. Source data are provided as a Source Data file.

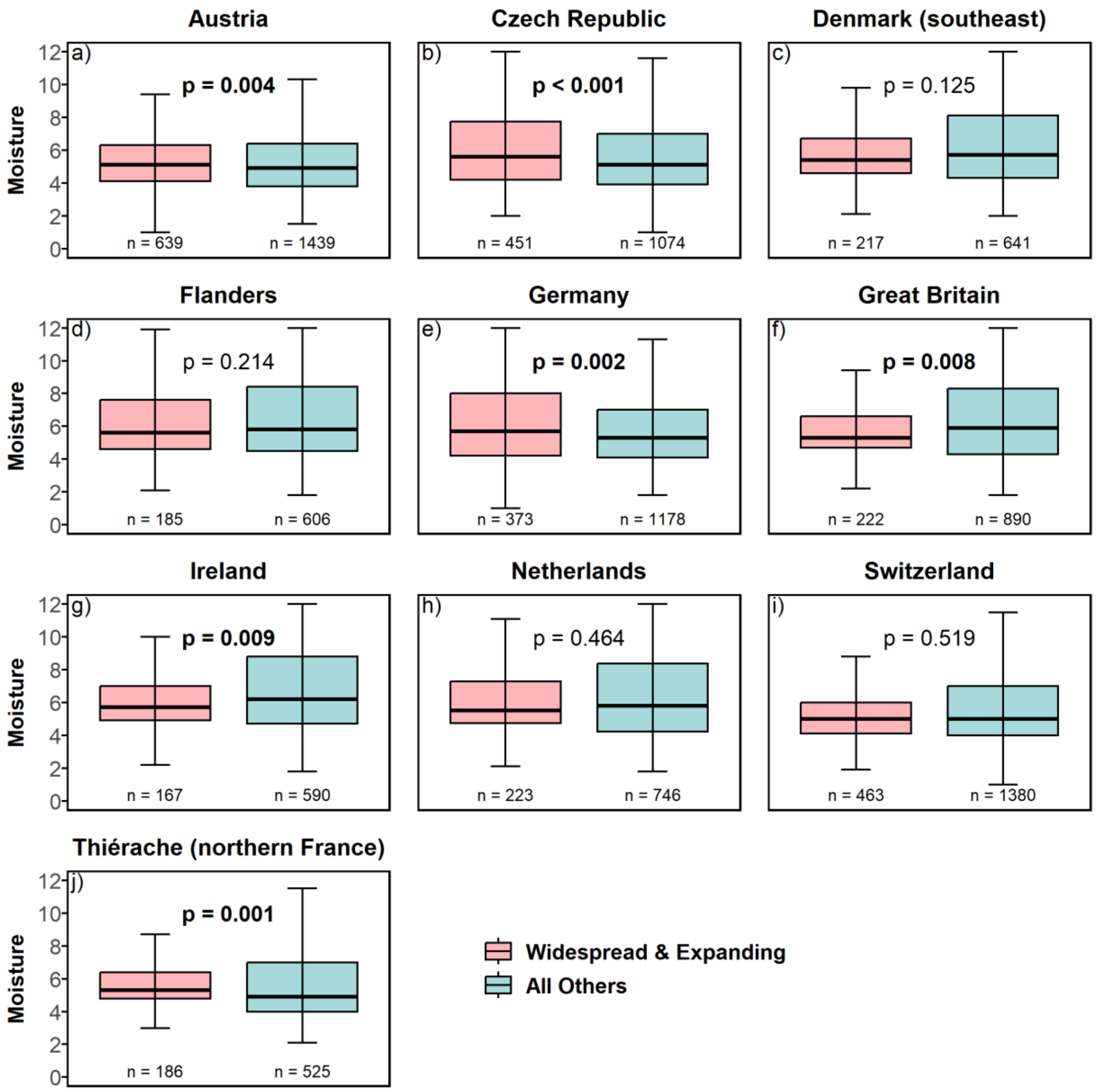

Fig. S9. Comparing Ellenberg indicator values for moisture between two species groups: " widespread and expanding " vs. all others for each of the 10 native regions (a-j). *P* values are from Mann-Whitney U test (see table S36), with significant values highlighted in bold. Sample sizes for each group in each native region are indicated by ‘n’ at the bottom of each box. Source data are provided as a Source Data file.

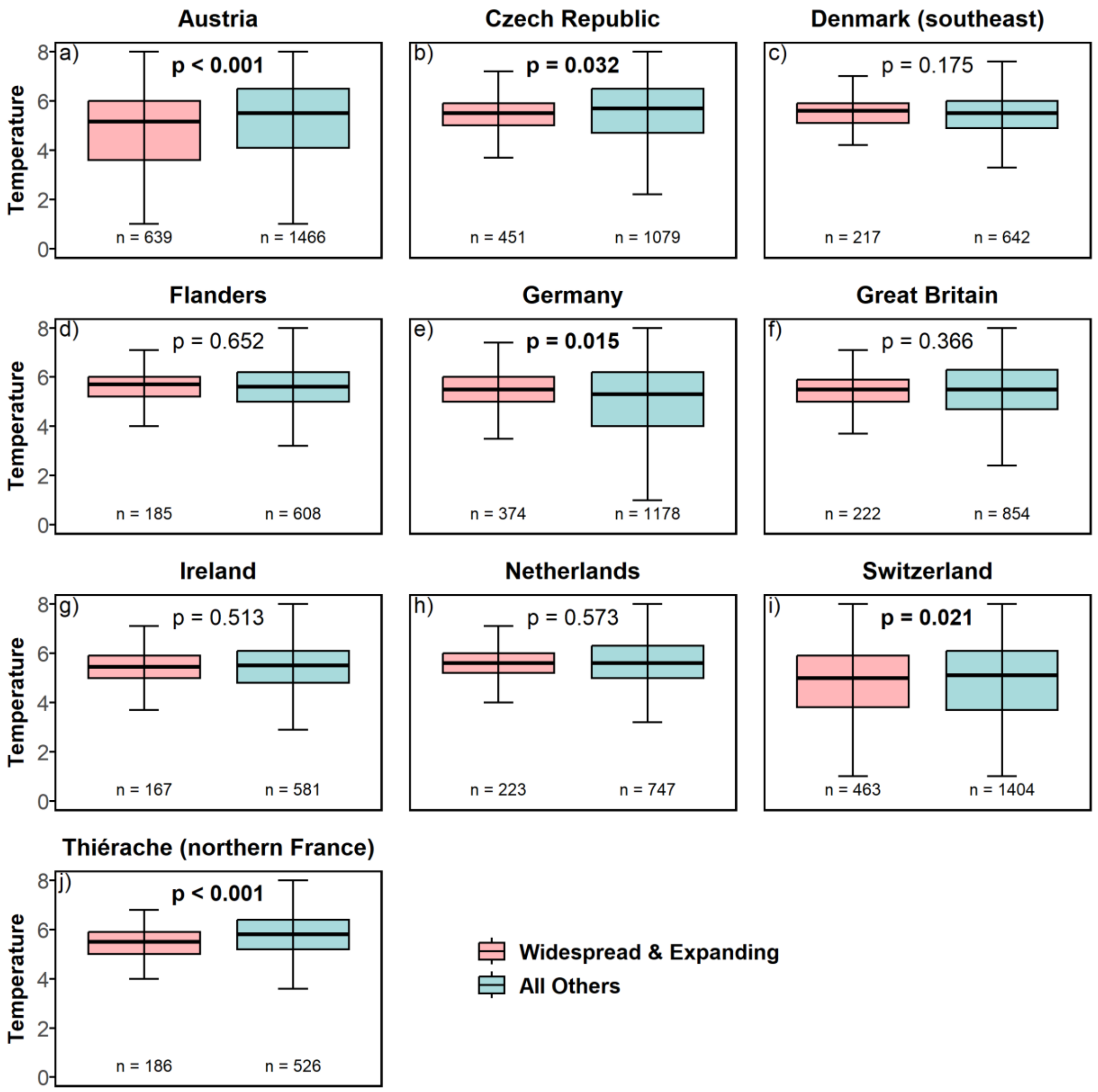

Fig. S10. Comparing Ellenberg indicator values for temperature between two species groups: " widespread and expanding " vs. all others for each of the 10 native regions (a-j). *P* values are from Mann-Whitney U test (see table S36), with significant values highlighted in bold. Sample sizes for each group in each native region are indicated by ‘n’ at the bottom of each box. Source data are provided as a Source Data file.

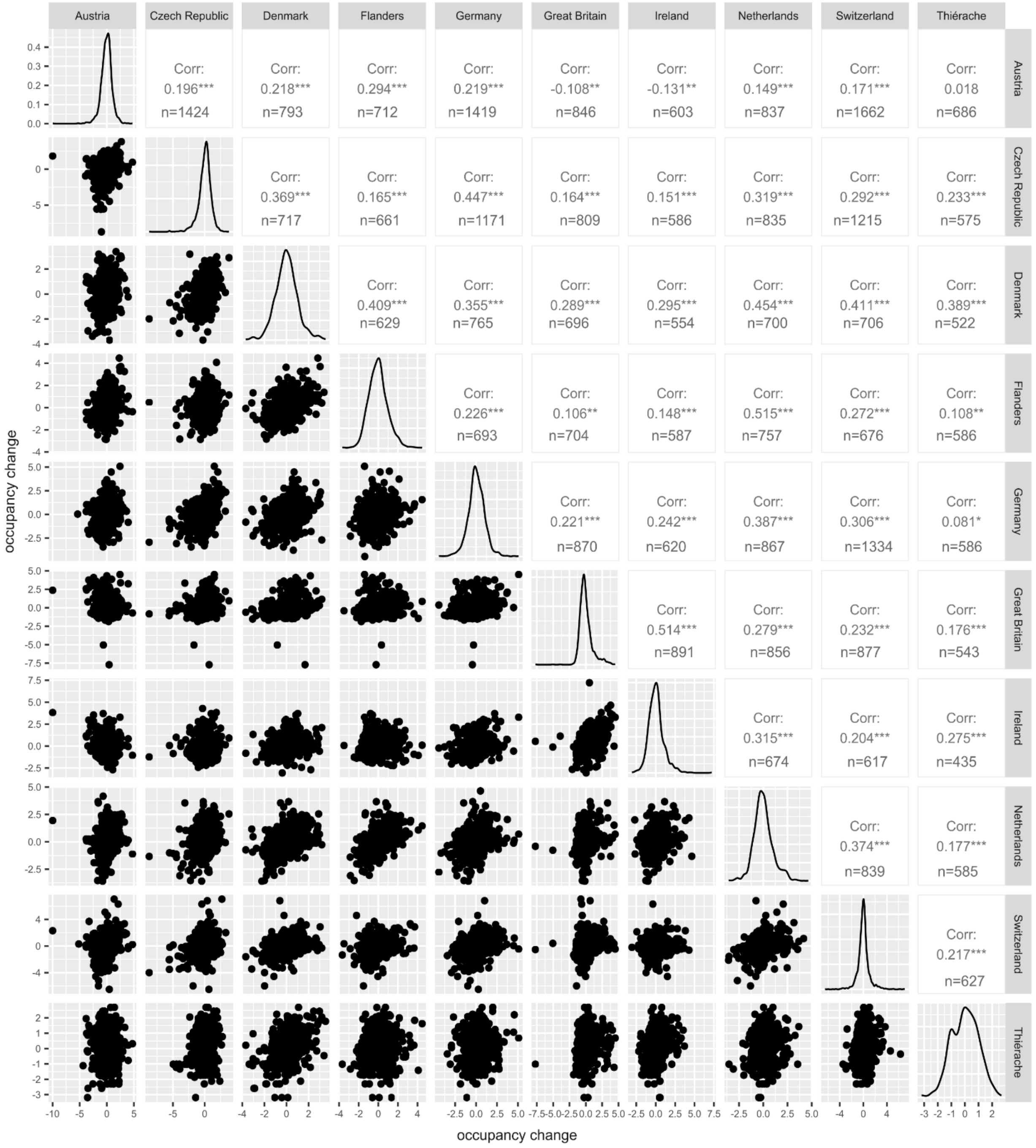

Fig. S11. Pairwise Pearson correlations of occupancy-change indices of species for each possible pair of the 10 native regions used in this study. The values in the boxes of the upper triangle are the Pearson correlation coefficients. The number of species shared between the regions is indicated as 'n' below the correlation value. Source data are provided as a Source Data file.

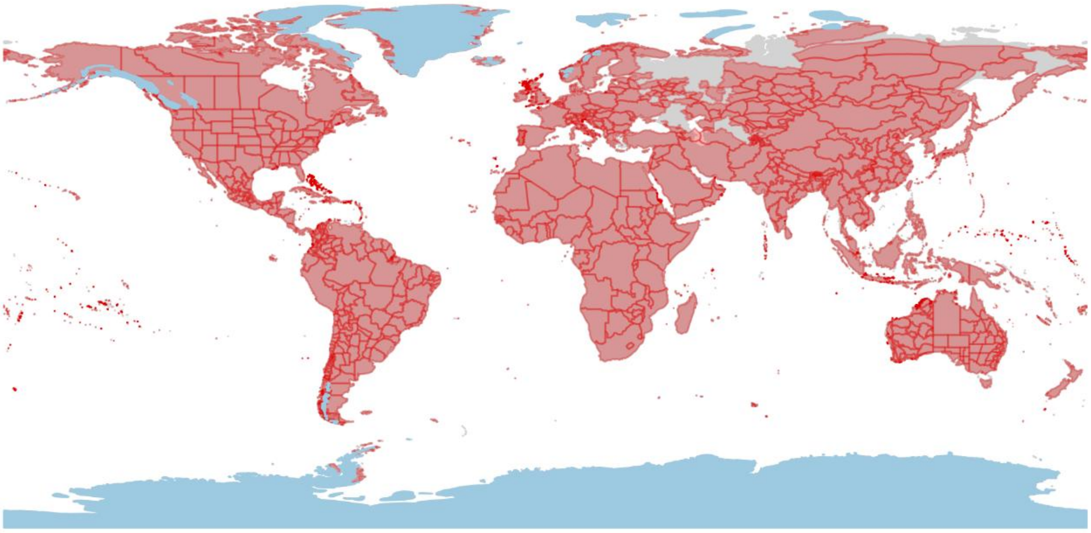

Fig. S12. Map showing the global coverage of non-overlapping regions (n = 920) in red for the most recent version of the GloNAF database. This database was used to quantify global naturalization success of species in this study. Terrestrial areas covered by ice sheets are indicated in blue. Regions without naturalization data are indicated in grey.

Table S1. Overview of the estimates of the two-part hurdle models. The Bernoulli part considers whether or not a species has become naturalized, and the zero-truncated count part considers the number of regions where a naturalized species has become naturalized. These models relate the two components of global naturalization success (naturalization incidence and extent) to occupancy in the early period (EO), the occupancy-change index (OC), woodiness of species (woody or non-woody) and their interaction for the ten native regions. To increase comparability, early occupancy was scaled to a mean of zero and a standard deviation of one. ***: P<0.001, **: P<0.01, *: P<0.05, **†:** marginally significant. All p-values are two-sided, and no adjustments for multiple comparisons were applied. The details of the hurdle model with exact p-values for each region are provided in **Tables S2-S11** separately. Source data are provided as a Source Data file.

	Austria	Czech Republic	Denmark (southeast)	Flanders (Belgium)	Germany	Great Britain	Ireland	Netherlands	Switzerland	Thiérache (northern France)
Bernoulli part										
Early occupancy (EO)	0.70***	1.09***	0.83***	1.61***	1.30***	1.19***	0.99***	1.09***	0.68***	1.10***
Occupancy change (OC)	0.16**	0.22**	0.62***	0.40†	0.09	0.09	0.01	0.61***	0.23***	0.17
Woodiness (W)	-0.15	-0.18	0.05	1.77	0.17	0.02	-0.13	-0.15	-0.09	0.04
EO × OC	-0.10	0.36***	-0.001	-0.04	0.08	0.04	-0.08	0.29	0.45***	0.18
EO × W	0.27†	0.14	0.36	2.47	0.42	0.59†	0.15	-0.36	0.29†	0.32
OC × W	0.11	-0.05	1.00*	2.11*	0.89**	0.42	0.03	0.32	0.15	0.49
EO × OC × W	0.15	0.29	0.17	3.06*	1.27**	0.43	-0.18	0.44	-0.14	0.48
Zero-truncated count part										
Early occupancy (EO)	0.38***	0.60***	0.49***	0.54***	0.67***	0.53***	0.53***	0.54***	0.39***	0.38***
Occupancy change (OC)	0.14***	0.08†	0.28***	0.26***	0.15***	0.25***	0.18*	0.48***	0.23***	0.03
Woodiness (W)	-0.54***	-0.25†	-0.51**	-0.37**	-0.31*	-0.49***	-0.41*	-0.42**	-0.24†	-0.42**
EO × OC	-0.04	0.12†	0.03	-0.01	-0.04	-0.002	0.001	0.26***	0.28***	0.04
EO × W	-0.04	-0.09	-0.17	-0.12	-0.01	0.13	0.04	0.20	-0.01	-0.17
OC × W	0.31†	0.42*	0.09	0.09	0.26*	-0.07	-0.02	0.03	-0.01	0.24
EO × OC × W	-0.05	-0.32*	0.24	-0.01	-0.18	-0.05	-0.25	-0.58**	-0.25†	-0.30

Table S2. Results of the hurdle models relating global naturalization success, separated into naturalization incidence (Bernoulli part) and the number of regions where naturalized species have become naturalized (zero-truncated count part), to occupancy in the early period, the occupancy-change index and woodiness of species (woody or non-woody) for Austria. To increase comparability, early occupancy was scaled to a mean of zero and a standard deviation of one. P-values < 0.05 are marked in bold. All p-values are two-sided, and no adjustments for multiple comparisons were applied. Source data are provided as a Source Data file.

Austria	Bernoulli part		Zero-truncated count part	
	Estimate ± SE	P	Estimate ± SE	P
Intercept	0.394 ± 0.047	**<0.001**	2.923 ± 0.083	**<0.001**
Early occupancy (EO)	0.704 ± 0.062	**<0.001**	0.384 ± 0.053	**<0.001**
Occupancy change (OC)	0.155 ± 0.050	**0.002**	0.141 ± 0.036	**<0.001**
Woodiness (W)	-0.146 ± 0.120	0.221	-0.536 ± 0.136	**<0.001**
EO × OC	-0.099 ± 0.063	0.115	-0.037 ± 0.034	0.275
EO × W	0.269 ± 0.161	0.094	-0.039 ± 0.125	0.752
OC × W	0.112 ± 0.157	0.474	0.309 ± 0.179	0.083
EO × OC ×W	0.148 ± 0.200	0.461	-0.053 ± 0.134	0.692
Log(theta)	-	-	-1.593 ± 0.116	**<0.001**

The pseudo R^2 by Cragg and Uhler (r2CU) was 0.143.

Table S3. Results of the hurdle models relating global naturalization success, separated into naturalization incidence (Bernoulli part) and the number of regions where naturalized species have become naturalized (zero-truncated count part) to occupancy in the early period, the occupancy-change index and woodiness of species (woody or non-woody) for the Czech Republic. To increase comparability, early occupancy was scaled to a mean of zero and a standard deviation of one. P-values < 0.05 are marked in bold. All p-values are two-sided, and no adjustments for multiple comparisons were applied. Source data are provided as a Source Data file.

Czech Republic	Bernoulli part		Zero-truncated count part	
	Estimate ± SE	P	Estimate ± SE	P
Intercept	0.794 ± 0.066	**<0.001**	2.469 ± 0.088	**<0.001**
Early occupancy (EO)	1.088 ± 0.093	**<0.001**	0.599 ± 0.058	**<0.001**
Occupancy change (OC)	0.224 ± 0.069	**0.001**	0.084 ± 0.046	0.066
Woodiness (W)	-0.179 ± 0.164	0.274	-0.246 ± 0.146	0.092
EO × OC	0.357 ± 0.096	**<0.001**	0.125 ± 0.064	0.050
EO × W	0.142 ± 0.238	0.551	-0.088 ± 0.155	0.569
OC × W	-0.049 ± 0.164	0.766	0.424 ± 0.166	**0.011**
EO × OC ×W	0.290 ± 0.253	0.252	-0.317 ± 0.158	**0.045**
Log(theta)	-	-	-1.446 ± 0.126	**<0.001**

The pseudo R^2 by Cragg and Uhler (r2CU) was 0.218.

Table S4. Results of the hurdle models relating global naturalization success, separated into naturalization incidence (Bernoulli part) and the number of regions where naturalized (zero-truncated count part) to occupancy in the early period, the occupancy-change index and woodiness of species (woody or non-woody) for southeastern Denmark. To increase comparability, early occupancy was scaled to a mean of zero and a standard deviation of one. P-values < 0.05 are marked in bold. All p-values are two-sided, and no adjustments for multiple comparisons were applied. Source data are provided as a Source Data file.

Southeastern Denmark	Bernoulli part		Zero-truncated count part	
	Estimate ± SE	P	Estimate ± SE	P
Intercept	1.617 ± 0.109	**<0.001**	3.393 ± 0.064	**<0.001**
Early occupancy (EO)	0.833 ± 0.113	**<0.001**	0.493 ± 0.057	**<0.001**
Occupancy change (OC)	0.621 ± 0.112	**<0.001**	0.279 ± 0.044	**<0.001**
Woodiness (W)	0.053 ± 0.387	0.892	-0.509 ± 0.168	**0.003**
EO × OC	-0.001 ± 0.123	0.992	0.028 ± 0.055	0.606
EO × W	0.364 ± 0.400	0.363	-0.170 ± 0.191	0.373
OC × W	0.996 ± 0.473	**0.035**	0.086 ± 0.145	0.554
EO × OC ×W	0.170 ± 0.507	0.738	0.244 ± 0.209	0.242
Log(theta)	-	-	-0.842 ± 0.092	**<0.001**

The pseudo R^2 by Cragg and Uhler (r2CU) was 0.258.

Table S5. Results of the hurdle models relating global naturalization success, separated into naturalization incidence (Bernoulli part) and the number of regions where naturalized species have become naturalized (zero-truncated count part) to occupancy in the early period, the occupancy-change index and woodiness of species (woody or non-woody) for Flanders (Belgium). To increase comparability, early occupancy was scaled to a mean of zero and a standard deviation of one. P-values < 0.05 are marked in bold. All p-values are two-sided, and no adjustments for multiple comparisons were applied. Source data are provided as a Source Data file.

Flanders (Belgium)	Bernoulli part		Zero-truncated count part	
	Estimate ± SE	P	Estimate ± SE	P
Intercept	2.300 ± 0.192	**<0.001**	3.494 ± 0.053	**<0.001**
Early occupancy (EO)	1.608 ± 0.273	**<0.001**	0.544 ± 0.048	**<0.001**
Occupancy change (OC)	0.403 ± 0.210	0.056	0.265 ± 0.048	**<0.001**
Woodiness (W)	1.773 ± 1.245	0.154	-0.368 ± 0.135	**0.006**
EO × OC	-0.044 ± 0.298	0.883	-0.013 ± 0.052	0.799
EO × W	2.467 ± 1.612	0.126	-0.123 ± 0.140	0.382
OC × W	2.113 ± 1.008	**0.036**	0.093 ± 0.128	0.470
EO × OC ×W	3.061 ± 1.289	**0.018**	-0.013 ± 0.146	0.927
Log(theta)	-	-	-0.528 ± 0.076	**<0.001**

The pseudo R^2 by Cragg and Uhler (r2CU) was 0.277.

Table S6. Results of the hurdle models relating global naturalization success, separated into naturalization incidence (Bernoulli part) and the number of regions where naturalized species have become naturalized (zero-truncated count part) to occupancy in the early period, the occupancy-change index and woodiness of species (woody or non-woody) for Germany. To increase comparability, early occupancy was scaled to a mean of zero and a standard deviation of one. P-values < 0.05 are marked in bold. All p-values are two-sided, and no adjustments for multiple comparisons were applied. Source data are provided as a Source Data file.

Germany	Bernoulli part		Zero-truncated count part	
	Estimate ± SE	P	Estimate ± SE	P
Intercept	0.907 ± 0.074	**<0.001**	2.602 ± 0.074	**<0.001**
Early occupancy (EO)	1.297 ± 0.109	**<0.001**	0.666 ± 0.050	**<0.001**
Occupancy change (OC)	0.085 ± 0.080	0.288	0.148 ± 0.043	**<0.001**
Woodiness (W)	0.170 ± 0.231	0.460	-0.308 ± 0.138	**0.026**
EO × OC	0.082 ± 0.111	0.461	-0.044 ± 0.037	0.239
EO × W	0.425 ± 0.344	0.216	-0.011 ± 0.139	0.939
OC × W	0.886 ± 0.317	**0.005**	0.261 ± 0.123	**0.034**
EO × OC ×W	1.266 ± 0.464	**0.006**	-0.177 ± 0.117	0.132
Log(theta)	-	-	-1.247 ± 0.108	**<0.001**

The pseudo R^2 by Cragg and Uhler (r2CU) was 0.278.

Table S7. Results of the hurdle models relating global naturalization success, separated into naturalization incidence (Bernoulli part) and the number of regions where naturalized species have become naturalized (zero-truncated count part) to occupancy in the early period, the occupancy-change index and woodiness of species (woody or non-woody) for Great Britain. To increase comparability, early occupancy was scaled to a mean of zero and a standard deviation of one. P-values < 0.05 are marked in bold. All p-values are two-sided, and no adjustments for multiple comparisons were applied. Source data are provided as a Source Data file.

Great Britain	Bernoulli part		Zero-truncated count part	
	Estimate ± SE	P	Estimate ± SE	P
Intercept	1.043 ± 0.083	**<0.001**	2.884 ± 0.775	**<0.001**
Early occupancy (EO)	1.190 ± 0.109	**<0.001**	0.531 ± 0.064	**<0.001**
Occupancy change (OC)	0.090 ± 0.085	0.290	0.251 ± 0.064	**<0.001**
Woodiness (W)	0.024 ± 0.267	0.928	-0.491 ± 0.147	**<0.001**
EO × OC	0.036 ± 0.094	0.704	-0.002 ± 0.040	0.964
EO × W	0.591 ± 0.353	0.094	0.127 ± 0.170	0.455
OC × W	0.415 ± 0.341	0.223	-0.068 ± 0.173	0.692
EO × OC ×W	0.428 ± 0.467	0.359	-0.051 ± 0.145	0.724
Log(theta)	-	-	-1.199 ± 0.111	**<0.001**

The pseudo R^2 by Cragg and Uhler (r2CU) was 0.279.

Table S8. Results of the hurdle models relating global naturalization success, separated into naturalization incidence (Bernoulli part) and the number of regions where naturalized species have become naturalized (zero-truncated count part) to occupancy in the early period, the occupancy-change index and woodiness of species (woody or non-woody) for Ireland. To increase comparability, early occupancy was scaled to a mean of zero and a standard deviation of one. P-values < 0.05 are marked in bold. All p-values are two-sided, and no adjustments for multiple comparisons were applied. Source data are provided as a Source Data file.

Ireland	Bernoulli part		Zero-truncated count part	
	Estimate ± SE	P	Estimate ± SE	P
Intercept	1.201 ± 0.097	**<0.001**	3.118 ± 0.085	**<0.001**
Early occupancy (EO)	0.990 ± 0.125	**<0.001**	0.525 ± 0.072	**<0.001**
Occupancy change (OC)	0.014 ± 0.097	0.882	0.182 ± 0.078	**0.020**
Woodiness (W)	-0.127 ± 0.235	0.588	-0.405 ± 0.167	**0.015**
EO × OC	-0.077 ± 0.113	0.495	0.001 ± 0.064	0.991
EO × W	0.149 ± 0.303	0.623	0.041 ± 0.182	0.820
OC × W	0.032 ± 0.234	0.892	-0.019 ± 0.171	0.914
EO × OC ×W	-0.182 ± 0.331	0.583	-0.254 ± 0.202	0.208
Log(theta)	-	-	-1.166 ± 0.122	**<0.001**

The pseudo R^2 by Cragg and Uhler (r2CU) was 0.201.

Table S9. Results of the hurdle models relating global naturalization success, separated into naturalization incidence (Bernoulli part) and the number of regions where naturalized species have become naturalized (zero-truncated count part) to occupancy in the early period, the occupancy-change index and woodiness of species (woody or non-woody) for the Netherlands. To increase comparability, early occupancy was scaled to a mean of zero and a standard deviation of one. P-values < 0.05 are marked in bold. All p-values are two-sided, and no adjustments for multiple comparisons were applied. Source data are provided as a Source Data file.

The Netherlands	Bernoulli part		Zero-truncated count part	
	Estimate ± SE	P	Estimate ± SE	P
Intercept	1.708 ± 0.117	**<0.001**	3.103 ± 0.056	**<0.001**
Early occupancy (EO)	1.092 ± 0.141	**<0.001**	0.538 ± 0.046	**<0.001**
Occupancy change (OC)	0.613 ± 0.142	**<0.001**	0.482 ± 0.058	**<0.001**
Woodiness (W)	-0.148 ± 0.268	0.580	-0.423 ± 0.137	**0.002**
EO × OC	0.288 ± 0.180	0.110	0.263 ± 0.070	**<0.001**
EO × W	-0.362 ± 0.336	0.280	0.197 ± 0.159	0.215
OC × W	0.325 ± 0.341	0.340	0.030 ± 0.165	0.857
EO × OC ×W	0.436 ± 0.436	0.317	-0.577 ± 0.217	**0.008**
Log(theta)	-	-	-0.756 ± 0.083	**<0.001**

The pseudo R^2 by Cragg and Uhler (r2CU) was 0.280.

Table S10. Results of the hurdle models relating global naturalization success, separated into naturalization incidence (Bernoulli part) and the number of regions where naturalized species have become naturalized (zero-truncated count part) to occupancy in the early period, the occupancy-change index and woodiness of species (woody or non-woody) for Switzerland. To increase comparability, early occupancy was scaled to a mean of zero and a standard deviation of one. P-values < 0.05 are marked in bold. All p-values are two-sided, and no adjustments for multiple comparisons were applied. Source data are provided as a Source Data file.

Switzerland	Bernoulli part		Zero-truncated count part	
	Estimate ± SE	P	Estimate ± SE	P
Intercept	0.279 ± 0.049	**<0.001**	2.411 ± 0.116	**<0.001**
Early occupancy (EO)	0.684 ± 0.059	**<0.001**	0.392 ± 0.054	**<0.001**
Occupancy change (OC)	0.229 ± 0.055	**<0.001**	0.233 ± 0.061	**<0.001**
Woodiness (W)	-0.086 ± 0.119	0.471	-0.244 ± 0.143	0.087
EO × OC	0.453 ± 0.075	**<0.001**	0.284 ± 0.076	**<0.001**
EO × W	0.287 ± 0.149	0.054	-0.014 ± 0.130	0.912
OC × W	0.146 ± 0.153	0.341	-0.009 ± 0.121	0.938
EO × OC ×W	-0.138 ± 0.203	0.495	-0.250 ± 0.140	0.074
Log(theta)	-	-	-1.810 ± 0.156	**<0.001**

The pseudo R^2 by Cragg and Uhler (r2CU) was 0.151.

Table S11. Results of the hurdle models relating global naturalization success, separated into naturalization incidence (Bernoulli part) and the number of regions where naturalized species have become naturalized (zero-truncated count part) to occupancy in the early period, the occupancy-change index and woodiness of species (woody or non-woody) for Thiérache (France). To increase comparability, early occupancy was scaled to a mean of zero and a standard deviation of one. P-values < 0.05 are marked in bold. All p-values are two-sided, and no adjustments for multiple comparisons were applied. Source data are provided as a Source Data file.

Thiérache (France)	Bernoulli part		Zero-truncated count part	
	Estimate ± SE	P	Estimate ± SE	P
Intercept	2.452 ± 0.175	**<0.001**	3.850 ± 0.054	**<0.001**
Early occupancy (EO)	1.102 ± 0.199	**<0.001**	0.381 ± 0.050	**<0.001**
Occupancy change (OC)	0.174 ± 0.152	0.251	0.029 ± 0.053	0.582
Woodiness (W)	0.035 ± 0.525	0.947	-0.424 ± 0.141	**0.003**
EO × OC	0.176 ± 0.161	0.274	0.038 ± 0.047	0.423
EO × W	0.318 ± 0.588	0.589	-0.168 ± 0.141	0.232
OC × W	0.491 ± 0.429	0.253	0.244 ± 0.173	0.160
EO × OC ×W	0.477 ± 0.499	0.340	-0.298 ± 0.183	0.104
Log(theta)	-	-	-0.551 ± 0.075	**<0.001**

The pseudo R^2 by Cragg and Uhler (r2CU) was 0.150.

Table S12. Results of the hurdle models relating global naturalization success, separated into naturalization incidence (Bernoulli part) and the number of regions where naturalized species have become naturalized (zero-truncated count part) to occupancy in the early period and the occupancy-change index for Austria. To increase comparability, early occupancy was scaled to a mean of zero and a standard deviation of one. The hurdle model consisted of a Bernoulli and a count (zero-truncated negative binomial) model. P-values < 0.05 are marked in bold. All p-values are two-sided, and no adjustments for multiple comparisons were applied. Source data are provided as a Source Data file.

Austria	Bernoulli part		Zero-truncated count part	
	Estimate ± SE	P	Estimate ± SE	P
Intercept	0.434 ± 0.046	**<0.001**	2.878 ± 0.085	**<0.001**
Early occupancy (EO)	0.761 ± 0.060	**<0.001**	0.361 ± 0.050	**<0.001**
Occupancy change (OC)	0.154 ± 0.049	**0.002**	0.145 ± 0.035	**<0.001**
EO × OC	-0.089 ± 0.063	0.158	-0.046 ± 0.033	0.16
Log(theta)	-	-	-1.617 ± 0.122	**<0.001**

The pseudo R^2 by Cragg and Uhler (r2CU) was 0.136.

Table S13. Results of the hurdle models relating global naturalization success, separated into naturalization incidence (Bernoulli part) and the number of regions where naturalized species have become naturalized (zero-truncated count part) to occupancy in the early period and the occupancy-change index for the Czech Republic. To increase comparability, early occupancy was scaled to a mean of zero and a standard deviation of one. The hurdle model consisted of a Bernoulli and a count (zero-truncated negative binomial) model. P-values < 0.05 are marked in bold. All p-values are two-sided, and no adjustments for multiple comparisons were applied. Source data are provided as a Source Data file.

Czech Republic	Bernoulli part		Zero-truncated count part	
	Estimate ± SE	P	Estimate ± SE	P
Intercept	0.752 ± 0.063	**<0.001**	2.453 ± 0.090	**<0.001**
Early occupancy (EO)	1.162 ± 0.089	**<0.001**	0.574 ± 0.056	**<0.001**
Occupancy change (OC)	0.206 ± 0.065	**0.001**	0.092 ± 0.043	**0.034**
EO × OC	0.397 ± 0.091	**<0.001**	0.083 ± 0.059	0.160
Log(theta)	-	-	-1.471 ± 0.131	**<0.001**

The pseudo R^2 by Cragg and Uhler (r2CU) was 0.216.

Table S14. Results of the hurdle models relating global naturalization success, separated into naturalization incidence (Bernoulli part) and the number of regions where naturalized species have become naturalized (zero-truncated count part) to occupancy in the early period and the occupancy-change index for southeastern Denmark. To increase comparability, early occupancy was scaled to a mean of zero and a standard deviation of one. The hurdle model consisted of a Bernoulli and a count (zero-truncated negative binomial) model. P-values < 0.05 are marked in bold. All p-values are two-sided, and no adjustments for multiple comparisons were applied. Source data are provided as a Source Data file.

southeastern Denmark	Bernoulli part		Zero-truncated count part	
	Estimate ± SE	P	Estimate ± SE	P
Intercept	1.645 ± 0.107	**<0.001**	3.363 ± 0.063	**<0.001**
Early occupancy (EO)	0.862 ± 0.110	**<0.001**	0.485 ± 0.056	**<0.001**
Occupancy change (OC)	0.667 ± 0.109	**<0.001**	0.265 ± 0.042	**<0.001**
EO × OC	-0.007 ± 0.120	0.954	0.036 ± 0.054	0.502
Log(theta)	-	-	-0.867 ± 0.095	**<0.001**

The pseudo R^2 by Cragg and Uhler (r2CU) was 0.236.

Table S15. Results of the hurdle models relating global naturalization success, separated into naturalization incidence (Bernoulli part) and the number of regions where naturalized species have become naturalized (zero-truncated count part) to occupancy in the early period and the occupancy-change index for Flanders (Belgium). To increase comparability, early occupancy was scaled to a mean of zero and a standard deviation of one. The hurdle model consisted of a Bernoulli and a count (zero-truncated negative binomial) model. P-values < 0.05 are marked in bold. All p-values are two-sided, and no adjustments for multiple comparisons were applied. Source data are provided as a Source Data file.

Flanders (Belgium)	Bernoulli part		Zero-truncated count part	
	Estimate ± SE	P	Estimate ± SE	P
Intercept	2.367 ± 0.196	**<0.001**	3.487 ± 0.050	**<0.001**
Early occupancy (EO)	1.588 ± 0.270	**<0.001**	0.526 ± 0.046	**<0.001**
Occupancy change (OC)	0.601 ± 0.204	**0.003**	0.255 ± 0.046	**<0.001**
EO × OC	0.171 ± 0.282	0.544	-0.028 ± 0.050	0.577
Log(theta)	-	-	-0.519 ± 0.078	**<0.001**

The pseudo R^2 by Cragg and Uhler (r2CU) was 0.263.

Table S16. Results of the hurdle models relating global naturalization success, separated into naturalization incidence (Bernoulli part) and the number of regions where naturalized species have become naturalized (zero-truncated count part) to occupancy in the early period and the occupancy-change index for Germany. To increase comparability, early occupancy was scaled to a mean of zero and a standard deviation of one. The hurdle model consisted of a Bernoulli and a count (zero-truncated negative binomial) model. P-values < 0.05 are marked in bold. All p-values are two-sided, and no adjustments for multiple comparisons were applied. Source data are provided as a Source Data file.

Germany	Bernoulli part		Zero-truncated count part	
	Estimate ± SE	P	Estimate ± SE	P
Intercept	0.977 ± 0.073	**<0.001**	2.584 ± 0.075	**<0.001**
Early occupancy (EO)	1.351 ± 0.108	**<0.001**	0.657 ± 0.048	**<0.001**
Occupancy change (OC)	0.156 ± 0.078	**0.045**	0.166 ± 0.040	**<0.001**
EO × OC	0.138 ± 0.108	0.202	-0.064 ± 0.035	0.069
Log(theta)	-	-	-1.273 ± 0.113	**<0.001**

The pseudo R^2 by Cragg and Uhler (r2CU) was 0.269.

Table S17. Results of the hurdle models relating global naturalization success, separated into naturalization incidence (Bernoulli part) and the number of regions where naturalized species have become naturalized (zero-truncated count part) to occupancy in the early period and the occupancy-change index for Great Britain. To increase comparability, early occupancy was scaled to a mean of zero and a standard deviation of one. The hurdle model consisted of a Bernoulli and a count (zero-truncated negative binomial) model. P-values < 0.05 are marked in bold. All p-values are two-sided, and no adjustments for multiple comparisons were applied. Source data are provided as a Source Data file.

Great Britain	Bernoulli part		Zero-truncated count part	
	Estimate ± SE	P	Estimate ± SE	P
Intercept	1.054 ± 0.081	**<0.001**	2.853 ± 0.075	**<0.001**
Early occupancy (EO)	1.278 ± 0.108	**<0.001**	0.527 ± 0.060	**<0.001**
Occupancy change (OC)	0.107 ± 0.082	0.188	0.233 ± 0.059	**<0.001**
EO × OC	0.063 ± 0.090	0.482	0.009 ± 0.038	0.817
Log(theta)	-	-	-1.185 ± 0.113	**<0.001**

The pseudo R^2 by Cragg and Uhler (r2CU) was 0.275.

Table S18. Results of the hurdle models relating global naturalization success, separated into naturalization incidence (Bernoulli part) and the number of regions where naturalized species have become naturalized (zero-truncated count part) to occupancy in the early period and the occupancy-change index for Ireland. To increase comparability, early occupancy was scaled to a mean of zero and a standard deviation of one. The hurdle model consisted of a Bernoulli and a count (zero-truncated negative binomial) model. P-values < 0.05 are marked in bold. All p-values are two-sided, and no adjustments for multiple comparisons were applied. Source data are provided as a Source Data file.

Ireland	Bernoulli part		Zero-truncated count part	
	Estimate ± SE	P	Estimate ± SE	P
Intercept	1.203 ± 0.093	**<0.001**	3.089 ± 0.082	**<0.001**
Early occupancy (EO)	1.022± 0.119	**<0.001**	0.528 ± 0.069	**<0.001**
Occupancy change (OC)	0.042 ± 0.091	0.641	0.180 ± 0.071	**0.011**
EO × OC	-0.080 ± 0.111	0.469	-0.004 ± 0.060	0.947
Log(theta)	-	-	-1.146 ± 0.124	**<0.001**

The pseudo R^2 by Cragg and Uhler (r2CU) was 0.195.

Table S19. Results of the hurdle models relating global naturalization success, separated into naturalization incidence (Bernoulli part) and the number of regions where naturalized species have become naturalized (zero-truncated count part) to occupancy in the early period and the occupancy-change index for the Netherlands. To increase comparability, early occupancy was scaled to a mean of zero and a standard deviation of one. The hurdle model consisted of a Bernoulli and a count (zero-truncated negative binomial) model. P-values < 0.05 are marked in bold. All p-values are two-sided, and no adjustments for multiple comparisons were applied. Source data are provided as a Source Data file.

The Netherlands	Bernoulli part		Zero-truncated count part	
	Estimate ± SE	P	Estimate ± SE	P
Intercept	1.755 ± 0.115	**<0.001**	3.085 ± 0.055	**<0.001**
Early occupancy (EO)	1.123 ± 0.140	**<0.001**	0.533 ± 0.046	**<0.001**
Occupancy change (OC)	0.721 ± 0.139	**<0.001**	0.449 ± 0.055	**<0.001**
EO × OC	0.401 ± 0.174	**0.021**	0.207 ± 0.068	**0.002**
Log(theta)	-	-	-0.773 ± 0.086	**<0.001**

The pseudo R^2 by Cragg and Uhler (r2CU) was 0.272.

Table S20. Results of the hurdle models relating global naturalization success, separated into naturalization incidence (Bernoulli part) and the number of regions where naturalized species have become naturalized (zero-truncated count part) to occupancy in the early period and the occupancy-change index for Switzerland. To increase comparability, early occupancy was scaled to a mean of zero and a standard deviation of one. The hurdle model consisted of a Bernoulli and a count (zero-truncated negative binomial) model. P-values < 0.05 are marked in bold. All p-values are two-sided, and no adjustments for multiple comparisons were applied. Source data are provided as a Source Data file.

Switzerland	Bernoulli part		Zero-truncated count part	
	Estimate ± SE	P	Estimate ± SE	P
Intercept	0.328 ± 0.047	**<0.001**	2.387 ± 0.117	**<0.001**
Early occupancy (EO)	0.750 ± 0.057	**<0.001**	0.384 ± 0.051	**<0.001**
Occupancy change (OC)	0.267 ± 0.054	**<0.001**	0.242 ± 0.057	**<0.001**
EO × OC	0.448 ± 0.074	**<0.001**	0.267 ± 0.070	**<0.001**
Log(theta)	-	-	-1.821 ± 0.162	**<0.001**

The pseudo R^2 by Cragg and Uhler (r2CU) was 0.150.

Table S21. Results of the hurdle models relating global naturalization success, separated into naturalization incidence (Bernoulli part) and the number of regions where naturalized species have become naturalized (zero-truncated count part) to occupancy in the early period and the occupancy-change index for Thiérache (France). To increase comparability, early occupancy was scaled to a mean of zero and a standard deviation of one. The hurdle model consisted of a Bernoulli and a count (zero-truncated negative binomial) model. P-values < 0.05 are marked in bold. All p-values are two-sided, and no adjustments for multiple comparisons were applied. Source data are provided as a Source Data file.

Thiérache (France)	Bernoulli part		Zero-truncated count part	
	Estimate ± SE	P	Estimate ± SE	P
Intercept	2.545 ± 0.176	**<0.001**	3.814 ± 0.053	**<0.001**
Early occupancy (EO)	1.168 ± 0.198	**<0.001**	0.354 ± 0.049	**<0.001**
Occupancy change (OC)	0.231 ± 0.146	0.113	0.019 ± 0.051	0.706
EO × OC	0.223 ± 0.154	0.146	0.016 ± 0.047	0.726
Log(theta)	-	-	-0.590 ± 0.078	**<0.001**

The pseudo R^2 by Cragg and Uhler (r2CU) was 0.130.

Table S22. Results of the Mann-Whitney U test, showing the relationship between early occupancy and woodiness (woody or non-woody). All p-values are two-sided, and no adjustments for multiple comparisons were applied. Source data are provided as a Source Data file.

Native regions	Median (woody)	Median (non-woody)	W	P
Austria	165	154	471065	0.765
Czech Republic	172	178	282528	0.592
Denmark	82.8	110	60500	0.111
Flanders	100	111	57989	0.504
Germany	691	724	242268	0.842
Great Britain	235	264	160263	0.723
Ireland	146	158	71282	0.759
The Netherlands	306	276	92242	0.577
Switzerland	181	170	463512	0.994
Thiérache	30	30	44791	0.867

Table S23. Results of the t-test, showing the relationship between occupancy change and woodiness (woody or non-woody). P-values < 0.05 are marked in bold. All p-values are two-sided, and no adjustments for multiple comparisons were applied. Source data are provided as a Source Data file.

Native regions	Mean ± SE (woody)	Mean ± SE (non- woody)	t-value	df	P
Austria	-0.081 ± 0.038	-0.058 ± 0.022	0.520	732.94	0.603
Czech Republic	0.321 ± 0.050	-0.048 ± 0.025	-6.614	539.17	**<0.001**
Denmark	0.245 ± 0.094	-0.051 ± 0.033	-2.962	165.77	**0.003**
Flanders	0.228 ± 0.097	-0.033 ± 0.035	-2.536	179.93	**0.012**
Germany	0.283 ± 0.055	-0.005 ± 0.025	-4.782	451.63	**<0.001**
Great Britain	-0.017 ± 0.054	-0.023 ± 0.029	-0.095	429.11	0.924
Ireland	-0.058 ± 0.086	0.039 ± 0.034	1.049	228.34	0.296
The Netherlands	0.214 ± 0.077	-0.040 ± 0.031	-3.073	248.52	**0.002**
Switzerland	0.058 ± 0.042	-0.001 ± 0.022	-1.245	727.62	0.214
Thiérache	0.210 ± 0.090	-0.055 ± 0.037	-2.710	171.79	**0.007**

Table S24. Variance explained (Pseudo R^2 values of Cragg and Uhler, r2CU) by different models.

Model I: Naturalization success ~ Early Occupancy

Model II: Naturalization success ~ Early Occupancy * Occupancy Change

Model III: Naturalization success ~ Early Occupancy *Woodiness

Model IV: Naturalization success ~ Early Occupancy * Occupancy Change*Woodiness

Native Regions	Pseudo R^2 values (by Cragg and Uhler), r2CU			
	Model I	Model II	Model III	Model IV
Austria	0.123	0.136	0.129	0.143
Czech Republic	0.206	0.216	0.203	0.218
Denmark (southeast)	0.152	0.236	0.158	0.258
Flanders	0.213	0.263	0.220	0.277
Germany	0.259	0.269	0.261	0.278
Great Britain	0.263	0.275	0.266	0.279
Ireland	0.187	0.195	0.191	0.201
The Netherlands	0.198	0.272	0.198	0.280
Switzerland	0.118	0.150	0.121	0.151
Thiérache	0.127	0.130	0.140	0.150

Table S25. Number of species present in at least 95 % of the total number of grid cells per region.

Regions	# total grid cells	# species present in all grid cells	# species present in more than 95% grid cells	# total native species used in analysis
Austria	2600	0	2	2419
Czech Republic	2551	0	0	1834
Denmark	263	0	5	921
Flanders	985	0	29	861
Germany	12024	0	65	1715
Great Britain	2852	0	0	1355
Ireland	1007	0	8	910
The Netherlands	1685	0	30	1115
Switzerland	1827	0	0	2307
Thiérache	129	0	0	775

Table S26. Results of the hurdle models relating global naturalization success, separated into naturalization incidence (Bernoulli part) and the number of regions where naturalized species have become naturalized (zero-truncated count part) to occupancy in the early period and the occupancy-change index for Great Britain considering 1987-1999 as the early time period and 2000-2019 as the later time period. To increase comparability, early occupancy was scaled to a mean of zero and a standard deviation of one. P-values < 0.05 are marked in bold. All p-values are two-sided, and no adjustments for multiple comparisons were applied. Source data are provided as a Source Data file.

Great Britain	Bernoulli part		Zero-truncated count part	
(1987-1999 and 2000-2019)	Estimate ± SE	P	Estimate ± SE	P
Intercept	0.989 ± 0.077	**<0.001**	2.831 ± 0.072	**<0.001**
Early occupancy (EO)	1.267 ± 0.102	**<0.001**	0.613 ± 0.055	**<0.001**
Occupancy change (OC)	-0.042 ± 0.084	0.613	0.286 ± 0.059	**<0.001**
EO × OC	- 0.011 ± 0.107	0.916	0.018 ± 0.056	0.744
Log(theta)	-	-	-1.126 ± 0.109	**<0.001**

The pseudo R^2 by Cragg and Uhler (r2CU) was 0.269.

Table S27. Results of the hurdle models relating global naturalization success, separated into naturalization incidence (Bernoulli part) and the number of regions where naturalized species have become naturalized (zero-truncated count part) to occupancy in the early period and the occupancy-change index for Great Britain considering 1970–1986 as the early time period and 1987–1999 as the later time period. To increase comparability, early occupancy was scaled to a mean of zero and a standard deviation of one. P-values < 0.05 are marked in bold. All p-values are two-sided, and no adjustments for multiple comparisons were applied. Source data are provided as a Source Data file.

Great Britain	Bernoulli part		Zero-truncated count part	
(1970-1986 and 1987-1999)	Estimate ± SE	P	Estimate ± SE	P
Intercept	1.044 ± 0.081	**<0.001**	2.841 ± 0.077	**<0.001**
Early occupancy (EO)	1.285 ± 0.109	**<0.001**	0.527 ± 0.059	**<0.001**
Occupancy change (OC)	0.114 ± 0.076	0.133	0.136 ± 0.063	**0.031**
EO × OC	0.067 ± 0.071	0.345	0.036 ± 0.034	0.284
Log(theta)	-	-	-1.217 ± 0.115	**<0.001**

The pseudo R^2 by Cragg and Uhler (r2CU) was 0.270.

Table S28. Results of the hurdle models relating global naturalization success, separated into naturalization incidence (Bernoulli part) and the number of regions where naturalized species have become naturalized (zero-truncated count part) to occupancy in the early period and the occupancy-change index for Great Britain considering 1970–1986 as the early time period and 2000-2019 as the later time period, considering only the occurrences grid cells that were considered by the original data source (Stroh et al. 2023) [7] as native. To increase comparability, early occupancy was scaled to a mean of zero and a standard deviation of one. P-values < 0.05 are marked in bold. All p-values are two-sided, and no adjustments for multiple comparisons were applied. Source data are provided as a Source Data file.

Great Britain (native only)	Bernoulli part		Zero-truncated count part	
(1970-1986 and 2000-2019)	Estimate ± SE	P	Estimate ± SE	P
Intercept	1.000 ± 0.077	**<0.001**	2.879 ± 0.076	**<0.001**
Early occupancy (EO)	1.200 ± 0.103	**<0.001**	0.482 ± 0.060	**<0.001**
Occupancy change (OC)	-0.025 ± 0.079	0.747	0.347 ± 0.074	**<0.001**
EO × OC	0.074 ± 0.087	0.397	-0.038 ± 0.043	0.381
Log(theta)	-	-	-1.195 ± 0.113	**<0.001**

The pseudo R^2 by Cragg and Uhler (r2CU) was 0.264.

Table S29. Results of the hurdle models relating global naturalization success, separated into naturalization incidence (Bernoulli part) and the number of regions where naturalized species have become naturalized (zero-truncated count part) to occupancy in the early period and the occupancy-change index for Ireland considering 1970–1986 as the early time period and 2000–2019 as the later time period. To increase comparability, early occupancy was scaled to a mean of zero and a standard deviation of one. P-values < 0.05 are marked in bold. All p-values are two-sided, and no adjustments for multiple comparisons were applied. Source data are provided as a Source Data file.

Ireland	Bernoulli part		Zero-truncated count part	
(1970-1986 and 2000–2019)	Estimate ± SE	P	Estimate ± SE	P
Intercept	1.275 ± 0.106	**<0.001**	3.134 ± 0.079	**<0.001**
Early occupancy (EO)	0.944 ± 0.140	**<0.001**	0.124 ± 0.087	0.153
Occupancy change (OC)	0.624 ± 0.112	**<0.001**	0.449 ± 0.077	**<0.001**
EO × OC	0.275 ± 0.149	0.064	0.134 ± 0.073	0.066
Log(theta)	-	-	-1.046 ± 0.119	**<0.001**

The pseudo R^2 by Cragg and Uhler (r2CU) was 0.210.

Table S30. Results of the hurdle models relating global naturalization success, separated into naturalization incidence (Bernoulli part) and the number of regions where naturalized species have become naturalized (zero-truncated count part) to occupancy in the early period and the occupancy-change index for Ireland considering 1970–1986 as the early time period and 1987-99 as the later time period. To increase comparability, early occupancy was scaled to a mean of zero and a standard deviation of one. P-values < 0.05 are marked in bold. All p-values are two-sided, and no adjustments for multiple comparisons were applied. Source data are provided as a Source Data file.

Ireland	Bernoulli part		Zero-truncated count part	
(1970-1986 and 1987-1999)	Estimate ± SE	P	Estimate ± SE	P
Intercept	1.310 ± 0.109	**<0.001**	3.103 ± 0.077	**<0.001**
Early occupancy (EO)	0.954 ± 0.143	**<0.001**	0.080 ± 0.078	0.307
Occupancy change (OC)	0.733 ± 0.113	**<0.001**	0.464 ± 0.069	**<0.001**
EO × OC	0.453 ± 0.152	**0.003**	0.299 ± 0.081	**<0.001**
Log(theta)	-	-	-0.994 ± 0.115	**<0.001**

The pseudo R^2 by Cragg and Uhler (r2CU) was 0.236.

Table S31. Results of the hurdle models relating global naturalization success, separated into naturalization incidence (Bernoulli part) and the number of regions where naturalized species have become naturalized (zero-truncated count part) to occupancy in the early period and the occupancy-change index for Ireland considering 1987-1999 as the early time period and 2000-2019 as the later time period, considering only the occurrences grid cells that were considered by the original data source (Stroh et al. 2023) [7] as native. To increase comparability, early occupancy was scaled to a mean of zero and a standard deviation of one. P-values < 0.05 are marked in bold. All p-values are two-sided, and no adjustments for multiple comparisons were applied. Source data are provided as a Source Data file.

Ireland (native only)	Bernoulli part		Zero-truncated count part	
(1987-1999 and 2000-2019)	Estimate ± SE	P	Estimate ± SE	P
Intercept	1.193 ± 0.092	**<0.001**	3.090 ± 0.082	**<0.001**
Early occupancy (EO)	1.006 ± 0.118	**<0.001**	0.520 ± 0.068	**<0.001**
Occupancy change (OC)	0.021± 0.090	0.812	0.166 ± 0.072	**0.022**
EO × OC	-0.057 ± 0.110	0.604	0.005 ± 0.060	0.930
Log(theta)	-	-	-1.151 ± 0.124	**<0.001**

The pseudo R^2 by Cragg and Uhler (r2CU) was 0.193.

Table S32. Results of the hurdle models relating global naturalization success, separated into naturalization incidence (Bernoulli part) and the number of regions where naturalized (zero-truncated count part) to occupancy in the early period and the occupancy-change index for the Netherlands considering the years before 2000 as the early time period and 2000-2024 as the later time period. To increase comparability, early occupancy was scaled to a mean of zero and a standard deviation of one. P-values < 0.05 are marked in bold. All p-values are two-sided, and no adjustments for multiple comparisons were applied. Source data are provided as a Source Data file.

The Netherlands	Bernoulli part		Zero-truncated count part	
(before 2000 and 2000-2024)	Estimate ± SE	P	Estimate ± SE	P
Intercept	1.706 ± 0.107	**<0.001**	3.099 ± 0.057	**<0.001**
Early occupancy (EO)	1.051 ± 0.125	**<0.001**	0.605 ± 0.046	**<0.001**
Occupancy change (OC)	0.627 ± 0.132	**<0.001**	0.325 ± 0.057	**<0.001**
EO × OC	0.293 ± 0.160	0.068	0.118 ± 0.069	0.085
Log(theta)	-	-	-0.824 ± 0.088	**<0.001**

Table S33. **Results of Mann-Whitney U test for Grime's CSR strategies comparing two species groups: "widespread and expanding (W&E)" vs. all others.** P-values < 0.05 are marked in bold. All p-values are two-sided, and no adjustments for multiple comparisons were applied. Source data are provided as a Source Data file.

	Austria	Czech Republic	Denmark (Southeast)	Flanders (Belgium)	Germany	Great Britain	Ireland	Netherlands	Switzerland	Thiérache (northern France)
C-Score										
Median (W&E)	23.990	31.245	33.043	32.131	28.488	27.983	27.286	30.012	26.327	30.542
Median (All others)	24.449	22.068	23.780	22.243	22.262	19.533	21.479	21.023	22.031	25.386
W	329247	157119	55945	45534	166325	73096	36964	77949	222006	43969
P	0.673	**<0.001**	**<0.001**	**<0.001**	**0.001**	**<0.001**	**<0.001**	**<0.001**	**0.009**	**0.001**
S-Score										
Median (W&E)	29.522	25.937	21.799	16.965	33.144	28.416	25.881	17.491	26.511	27.813
Median (All others)	27.240	29.480	22.592	24.539	26.011	28.153	26.546	26.463	29.043	20.319
W	318344	206435	75275	67364	177740	94762	49262	107220	246292	47915
P	0.120	**0.022**	0.200	**0.011**	0.090	0.183	0.380	**0.005**	0.652	0.078
R-Score										
Median (W&E)	37.286	35.836	41.241	42.541	32.507	39.607	40.194	41.134	36.034	39.093
Median (All others)	39.638	38.957	42.129	42.906	39.146	39.881	42.422	41.830	38.813	44.491
W	350327	199888	76559	61264	208615	92307	49397	96809	253633	59983
P	0.080	0.207	0.094	0.689	**0.003**	0.488	0.352	0.893	0.168	**0.002**

Table S34. Results of Mann-Whitney U test for Ellenberg indicators comparing two species groups: "widespread and expanding (W&E)" vs. all others. P-values < 0.05 are marked in bold. All p-values are two-sided, and no adjustments for multiple comparisons were applied. Source data are provided as a Source Data file.

	Austria	Czech Republic	Denmark (Southeast)	Flanders (Belgium)	Germany	Great Britain	Ireland	Netherlands	Switzerland	Thiérache (northern France)
Light										
Median (W&E)	7.3	7.0	7.0	7.1	7.1	7.0	7.0	7.2	7.2	7.0
Median (All others)	7.5	7.3	7.3	7.3	7.5	7.5	7.5	7.3	7.5	7.3
W	515639	283595	80393	65351	257785	128964	64818	93159	357695	63643
P	**<0.001**	**<0.001**	**<0.001**	**0.001**	**<0.001**	**<0.001**	**<0.001**	**0.006**	**<0.001**	**<0.001**
Nutrients										
Median (W&E)	4.0	5.0	5.6	5.8	4.6	5.0	5	5.6	4.3	5.3
Median (All others)	3.3	3.3	4.0	4.5	3.3	3.7	4	4.0	3.0	4.7
W	379518	155754	40103	34903	154361	69089	35871	81221	240766	35773
P	**<0.001**	**<0.001**	**<0.001**	**<0.001**	**<0.001**	**<0.001**	**<0.001**	**<0.001**	**<0.001**	**<0.001**
Moisture										
Median (W&E)	5.1	5.6	5.4	5.6	5.7	5.3	5.7	5.5	5	5.3
Median (All others)	4.9	5.1	5.7	5.8	5.3	5.9	6.2	5.8	5	4.9
W	423754	210270	74393	59436	196698	110111	55794	85867	325857	41008
P	**0.004**	**<0.001**	0.125	0.214	**0.002**	**0.008**	**0.009**	0.464	0.519	**0.001**
Temperature										
Median (W&E)	5.2	5.5	5.6	5.7	5.5	5.5	5.5	5.6	5.0	5.5
Median (All others)	5.5	5.7	5.5	5.6	5.3	5.6	5.5	5.6	5.1	5.8
W	538020	260155	65371	55011	201985	98520	50121	48659	348277	60970
P	**<0.001**	**0.032**	0.175	0.652	**0.015**	0.366	0.513	0.573	**0.021**	**<0.001**

Table S35. Pearson correlations of native range size with early occupancy and occupancy change for the species in the 10 native regions. Native range size was quantified as the total number of botanical countries (i.e. TDWG3 regions) where the species are native to according to the POWO database. P-values < 0.05 are marked in bold. All p-values are two-sided, and no adjustments for multiple comparisons were applied.

Native region	No. species	Early occupancy		Occupancy change	
		r	P	r	P
Austria	2419	0.329	**<0.001**	0.130	**<0.001**
Czech Republic	1834	0.374	**<0.001**	-0.028	0.230
Denmark (southeast)	921	0.251	**<0.001**	0.011	0.738
Flanders	861	0.239	**<0.001**	0.0349	0.314
Germany	1715	0.404	**<0.001**	-0.078	**0.001**
Great Britain	1355	0.309	**<0.001**	-0.025	0.365
Ireland	910	0.239	**<0.001**	-0.092	**0.005**
The Netherlands	1115	0.324	**<0.001**	-0.036	0.230
Switzerland	2307	0.303	**<0.001**	-0.091	**<0.001**
Thiérache	775	0.182	**<0.001**	0.0216	0.549

Table S36. Number of species present in different numbers of the ten native regions. A detailed record of the presence and absence of all 3,920 species across these regions can be found in repository (Figshare; https://doi.org/10.6084/m9.figshare.25487209).

No. of regions where species is present	No. of species
10	288
9	186
8	144
7	130
6	165
5	218
4	356
3	487
2	685
1	1261

References

1. University of Vienna, I. f. B., Research Group for Plant Biogeography. *Floristische Kartierung Österreichs - Mapping the Flora of Austria. Occurrence dataset.*
2. Wild, J. et al. Plant distribution data for the Czech Republic integrated in the Pladias database. *Preslia* **91**(1), 1-24 (2019).
3. Nielsen, T. F., Sand-Jensen, K., Dornelas, M. & Bruun, H. H. More is less: net gain in species richness, but biotic homogenization over 140 years. *Ecology Letters* **22**(10), 1650-1657 (2019).
4. Van Landuyt, W. et al. *Atlas van de flora van Vlaanderen en het Brussels Gewest: inleiding*, in *Atlas van de flora van Vlaanderen en het Brussels Gewest.* 2006, Nationale Plantentuin van België. p. 9-13.
5. Eichenberg, D. et al. Widespread decline in Central European plant diversity across six decades. *Global Change Biology* **27**(5), 1097-1110 (2021).
6. Hill, M. O. Local frequency as a key to interpreting species occurrence data when recording effort is not known. *Methods in Ecology and Evolution* **3**(1), 195-205 (2012).
7. Stroh, P. A. et al. Plant atlas 2020: mapping changes in the distribution of the British and Irish flora (Princeton University Press, 2023)
8. Preston, C. D., Pearman, D. & Dines, T. D. New atlas of the British & Irish flora (Oxford University Press, 2002)
9. NDFF. NDFF Verspreidingsatlas. *URL: http://verspreidingsatlas.nl/* (2015).
10. Flora, I. The national data and information center on the Swiss flora. *URL: https://www.infoflora.ch/de/ [last accessed: 5 July 2019]* (2021).
11. Van Calster, H. et al. Unexpectedly high 20th century floristic losses in a rural landscape in northern France. *Journal of Ecology* **96**(5), 927-936 (2008).

12. Kattge, J. et al. TRY plant trait database–enhanced coverage and open access. *Global change biology* **26**(1), 119-188 (2020).
13. Taylor, A. et al. The contribution of plant life and growth forms to global gradients of vascular plant diversity. *New Phytologist* **240**(4), 1548-1560 (2023).
14. Henniges, M. C. et al. A taxonomic, genetic and ecological data resource for the vascular plants of Britain and Ireland. *Scientific Data* **9**(1), 1 (2022).
15. Weigelt, P., König, C. & Kreft, H. GIFT–A global inventory of floras and traits for macroecology and biogeography. *Journal of Biogeography* **47**(1), 16-43 (2020).
16. Denelle, P. & Weigelt, P. GIFT: Access to the Global Inventory of Floras and Traits (GIFT). *R package version 1.3.2, https://CRAN.R-project.org/package=GIFT* (2024).
17. Luo, A. et al. Spatio-temporal patterns in the woodiness of flowering plants. *Global Ecology and Biogeography* **32**(3), 384-396 (2023).
18. Zanne, A. E. et al. Three keys to the radiation of angiosperms into freezing environments. *Nature* **506**(7486), 89-92 (2014).
19. Brummitt, R. K., Pando, F., Hollis, S. & Brummitt, N. World Geographical Scheme for Recording Plant Distributions (International Working Group on Taxonomic Databases for Plant Sciences (TDWG), Brussels, 2001)
20. Grime, J. P. Vegetation classification by reference to strategies. *Nature* **250** (5461), 26-31 (1974).
21. Ellenberg, H. Zeigerwerte der Gefäßpflanzen Mitteleuropas (Indicator values of vascular plants in Central Europe). Scripta Geobotanica **9**, 1– 97 (1974).
22. Guo, Wen-Yong et al. The role of adaptive strategies in plant naturalization. *Ecology letters* **21**, 1380-1389 (2018).
23. Pierce, S. et al. A global method for calculating plant CSR ecologicalstrategies applied across biomes world-wide. *Functional Ecology* **31**, 444–457 (2017).

24. Tichý, L. et al. Ellenberg-type indicator values for European vascular plant species. *Journal of Vegetation Science*, **34** (1), e13168 (2024).

25. Chytrý M., et al. Pladias Database of the Czech Flora and Vegetation. *Preslia **93**, 1–87* (2021).

26. Telfer, M. G., Preston, C. & Rothery, P. A general method for measuring relative change in range size from biological atlas data. *Biological conservation* **107**(1), 99-109 (2002).
