# Supplementary material for: Many plants naturalized as aliens abroad have also become more common within their native regions
Source: Nat Commun. 2025 Sep 5;16:8227. doi: 10.1038/s41467-025-63293-6 (PMC12413455; doi:10.1038/s41467-025-63293-6)
Supplement: Supplementary file 2 — Reporting Summary [file 41467_2025_63293_MOESM2_ESM.pdf]

Corresponding author(s): Rashmi Paudel
Last updated by author(s): Jul 15, 2025

Reporting Summary

Nature Portfolio wishes to improve the reproducibility of the work that we publish. This form provides structure for consistency and transparency in reporting. For further information on Nature Portfolio policies, see our Editorial Policies and the Editorial Policy Checklist.

Statistics

For all statistical analyses, confirm that the following items are present in the figure legend, table legend, main text, or Methods section.

n/a	Confirmed	
☐	☒	The exact sample size (n) for each experimental group/condition, given as a discrete number and unit of measurement
☒	☐	A statement on whether measurements were taken from distinct samples or whether the same sample was measured repeatedly
☐	☒	The statistical test(s) used AND whether they are one- or two-sided<br>*Only common tests should be described solely by name; describe more complex techniques in the Methods section.*
☐	☒	A description of all covariates tested
☐	☒	A description of any assumptions or corrections, such as tests of normality and adjustment for multiple comparisons
☐	☒	A full description of the statistical parameters including central tendency (e.g. means) or other basic estimates (e.g. regression coefficient) AND variation (e.g. standard deviation) or associated estimates of uncertainty (e.g. confidence intervals)
☐	☒	For null hypothesis testing, the test statistic (e.g. F, t, r) with confidence intervals, effect sizes, degrees of freedom and P value noted<br>*Give P values as exact values whenever suitable.*
☒	☐	For Bayesian analysis, information on the choice of priors and Markov chain Monte Carlo settings
☒	☐	For hierarchical and complex designs, identification of the appropriate level for tests and full reporting of outcomes
☐	☒	Estimates of effect sizes (e.g. Cohen's d, Pearson's r), indicating how they were calculated

Our web collection on statistics for biologists contains articles on many of the points above.

Software and code

Policy information about availability of computer code

Data collection	As data were collected from scientific publications, online plant atlases and by contacting curators species distribution databases, no software was used for data collection. The data were entered in Excel spreadsheets.
Data analysis	Data were analyzed using R version 4.2.3, following the code provided in the repository (https://doi.org/10.6084/m9.figshare.25487209). Species names were harmonized using the TNRS package (version 0.3.3) and rWCVP (version 1.0.3), based on the taxonomic backbone of the World Checklist of Vascular Plants (version 11). Hurdle models were performed using the pscl package (version 1.5.5). The model consisted of a generalized linear model (GLM) with a Bernoulli distribution, and a GLM with a zero-truncated negative binomial distribution.

For manuscripts utilizing custom algorithms or software that are central to the research but not yet described in published literature, software must be made available to editors and reviewers. We strongly encourage code deposition in a community repository (e.g. GitHub). See the Nature Portfolio guidelines for submitting code & software for further information.

Data

Policy information about availability of data

All manuscripts must include a data availability statement. This statement should provide the following information, where applicable:

- Accession codes, unique identifiers, or web links for publicly available datasets
- A description of any restrictions on data availability
- For clinical datasets or third party data, please ensure that the statement adheres to our policy

All the data required to reproduce the results can be downloaded through https://figshare.com/s/09e704abbf25d6218692

Research involving human participants, their data, or biological material

Policy information about studies with human participants or human data. See also policy information about sex, gender (identity/presentation), and sexual orientation and race, ethnicity and racism.

Reporting on sex and gender	not applicable
Reporting on race, ethnicity, or other socially relevant groupings	not applicable
Population characteristics	not applicable
Recruitment	not applicable
Ethics oversight	not applicable

Note that full information on the approval of the study protocol must also be provided in the manuscript.

Field-specific reporting

Please select the one below that is the best fit for your research. If you are not sure, read the appropriate sections before making your selection.

☐ Life sciences ☐ Behavioural & social sciences ☒ Ecological, evolutionary & environmental sciences

For a reference copy of the document with all sections, see nature.com/documents/nr-reporting-summary-flat.pdf

Ecological, evolutionary & environmental sciences study design

All studies must disclose on these points even when the disclosure is negative.

Study description	We collected data on grid-cell occupancy of vascular plant species for 10 European regions that had such data for at least two periods, spanning decades to more than one century. For each of these regions, we calculated an occupancy-change index for each native species and tested how this change index along with occupancy in the early period relate to global naturalization. Specifically, we tested whether the species that have increased their occupancy at home are the same species that also have become widely naturalized as aliens abroad.
Research sample	To our knowledge, our sample of change in occupancy of species over the decades to century in their native range, mostly cover the available data on grid-cell occupancy of vascular plants for at least two periods.
Sampling strategy	Our initial sample includes all the species, from each of the 10 European regions, that have data on grid-cell occupancy for at least two time periods and that are native to the respective regions. Then, we used the World Checklist of Vascular Plants (WCVP) taxonomic backbone to harmonize the taxonomic names and the taxa without accepted names were removed. Additionally, if multiple species in the original dataset were assigned to the same accepted name in WCVP, we kept the one that had the largest number of occupied grid cells. During the occupancy-change index calculation, we excluded species occupying fewer than five grid cells during the early period. This was done because when we visualized the data using scatterplots, species with low occupancy proportions in the early period deviated from the linear relationship.
Data collection	Data from the Global Naturalized Alien Flora database (version 2.0) was extracted by Amy Davis in January 2024. The used version (version 11) of the data from World Checklist of Vascular Plants (WCVP, which is integrated in POWO) was downloaded by Mark van Kleunen in April 2023.<br><br>Data on native range occupancy over time was collected from scientific publications, online plant atlases and by contacting curators of plant species distribution databases, by Mark van Kleunen and Rashmi Paudel, between October 2022 and May 2023. Hans van Calster and Guillaume Decocq provided the data on species occupancy for Thiérache (northern France). Milan Chytrý, Jiří Danihelka, Zdeněk Kaplan, Jan Wild and Petr Pyšek provided the data for the Czech Republic. For Austria, Luise Ehrendorfer-Schratt provided the data on occupancy.

From October 2022 to April 2023, Rashmi Paudel, first matched the original species names with WCVP version 10 for taxonomic harmonization. This was done in R version 4.2.3, initially with the TNRS package version 0.3.3, and later, after it became available, the rWCVP package version 1.0.3. Then, the obtained datasets were aligned with the global naturalization data available from the Global Naturalized Alien Flora database. In April 2023, all the species from each 10 regions were updated to version 11 of WCVP.

From June 2024 to September 2024, data on woodiness was collected by Rashmi Paudel and Mark van Kleunen. These data were collected from scientific publications, online databases and by internet searches.

From February 2025 to May 2025, data on Ellenberg indicators was collected by Rashmi Paudel and Mark van Kleunen, from scientific publications and online databases. Kun Guo, Simon Pierce and Wen-Yong Guo provide data on Grime's CSR strategies.

Timing and spatial scale	The data on occupancy frequencies in the different regions covers the period 1880 to 2022. The species analysed in our research cover the European native regions and our data on naturalization covers the global status on naturalization of each species from each region.
Data exclusions	All species with complete data (except the selection mentioned in Sampling strategy) were included in the analyses.
Reproducibility	The final datasets and R code for reproducing results are available in a figshare repository (https://figshare.com/s/09e704abbf25d6218692).
Randomization	not applicable
Blinding	This is not applicable, because for data acquisition and analysis we used all available occurrence data for each of the 10 regions.

Did the study involve field work? ☐ Yes ☒ No

Reporting for specific materials, systems and methods

We require information from authors about some types of materials, experimental systems and methods used in many studies. Here, indicate whether each material, system or method listed is relevant to your study. If you are not sure if a list item applies to your research, read the appropriate section before selecting a response.

Materials & experimental systems

n/a	Involved in the study
☒	☐ Antibodies
☒	☐ Eukaryotic cell lines
☒	☐ Palaeontology and archaeology
☒	☐ Animals and other organisms
☒	☐ Clinical data
☒	☐ Dual use research of concern
☒	☐ Plants

Methods

n/a	Involved in the study
☒	☐ ChIP-seq
☒	☐ Flow cytometry
☒	☐ MRI-based neuroimaging

Plants

Seed stocks	not applicable
Novel plant genotypes	not applicable
Authentication	not applicable
